# An FPGA-Based Event-Timing Front-End for Time-Resolved Sensing with Dual-Mode Experimental Characterization

**DOI:** 10.3390/s26103268

**Published:** 2026-05-21

**Authors:** Juan Núñez, Rafaella Fiorelli

**Affiliations:** Instituto de Microelectrónica de Sevilla, IMSE-CNM (CSIC/Universidad de Sevilla), Américo Vespucio 28, 41092 Sevilla, Spain; fiorelli@csic.es

**Keywords:** FPGA TDC, time-to-digital converter, event timing, time-resolved sensing, SPAD-based sensing workflows, coarse–fine timestamping, event qualification, timing front-end, detector-independent timing subsystem

## Abstract

This work presents an FPGA-based edge-event timing front-end for time-resolved sensing and event-driven measurement scenarios. The proposed design is intended as a detector-independent timing subsystem whose architectural choices are motivated by constraints that are common in single-photon avalanche diode (SPAD)-based and other asynchronous time-resolved sensing workflows, including event trustworthiness, dead-time sensitivity, and constrained downstream readout. Rather than treating the implementation as an isolated interpolation macro, this work evaluates it as an experimentally observable timing subsystem that combines carry-chain-based fine interpolation, coarse–fine timestamp formation, explicit event-quality assessment, dead-time-aware handling, and lightweight host-visible export. The experimental validation is organized around two complementary modes. An internal ILA-based mode is used to verify coherent front-end behavior under MHz-range short-pulse excitation, while a UART-based campaign identifies practical host-visible operating regions through baseline, repeatability, pulse-width, safe-versus-aggressive, and intermediate frequency-sweep experiments. The results identify a safe export-compatible operating point, a more exploratory high-rate regime, and an experimentally interpretable transition between them that, while not strictly monotonic in all metrics, does not exhibit catastrophic degradation across the explored frequency range. Taken together, the measurements indicate that the proposed architecture is best understood not as a best-case standalone time-to-digital (TDC) benchmark but as an experimentally characterized timing front-end whose practical behavior can be interpreted across complementary internal and export-visible operating regimes.

## 1. Introduction

Precise event timing is a foundational requirement in a wide class of modern sensing and instrumentation systems. In many architectures of practical and scientific interest, the most relevant observable is not the amplitude of a slowly varying analog signal but the exact arrival time of one or more transitions. This timing-centric view arises naturally in time-of-flight (ToF) ranging, photon-counting instrumentation, coincidence measurements, pulsed radiation sensing, asynchronous trigger chains, and fast biomedical or laboratory front-ends. Time-to-digital converters (TDCs), therefore, remain central building blocks in time-domain measurement systems, and their design principles, implementation trade-offs, and error sources are well established in both foundational monographs and recent surveys [1,2].

The importance of timing extraction becomes even more evident in time-resolved sensing. In single-photon avalanche diode (SPAD) imagers and related optical timing systems, the usefulness of the detector is inseparable from the timestamping or histogramming chain that follows each detected event [3,4]. This applies to direct and indirect ToF imaging, fluorescence lifetime imaging microscopy (FLIM), time-correlated single-photon counting (TCSPC), fast depth sensing, and other photon-efficient low-light instrumentation workflows [5,6]. Recent sensor-side work on SPAD-based LiDAR arrays, direct ToF architectures, TDC-sharing readout strategies, correlation-assisted pixels, short-pulse demodulator pixels, and stacked low-light SPAD imagers further illustrates how tightly detector architecture and timing electronics are coupled in practical systems [7,8,9,10,11,12,13]. Similar considerations also apply beyond optical sensing whenever asynchronous physical events must be converted into trustworthy digital timing information under constrained laboratory conditions.

A practical consequence of this context is that a timing front-end should not be evaluated only by nominal interpolation capability. In sensing-oriented instrumentation, the useful performance of the front-end depends simultaneously on several layers: the quality of the event interface, the robustness of the fine-time extraction stage, the validity of the resulting timestamp, the treatment of abnormal events, the available export bandwidth, and the reproducibility of the complete acquisition workflow. This broader perspective is consistent with instrumentation-oriented reviews spanning detector metrology, TCSPC electronics, and FLIM microsystems, all of which emphasize that timing quality must be interpreted together with the acquisition chain that supports it [14,15,16].

Field-programmable gate arrays (FPGAs) are attractive platforms for implementing this type of front-end because they offer flexibility, short development cycles, relatively low cost, and access to native fast-carry resources that can be exploited for within-clock-cycle interpolation. More broadly, timing front-ends for SPAD-based systems also draw on insights from the detector and readout literature, from early timing demonstrations with discrete SPADs to later reviews of array-level device and electronics behavior [17,18,19,20,21]. FPGA-based TDCs have therefore been studied for more than a decade, and the field has matured to the point that a new contribution in this area can no longer be justified simply by showing that a delay-line TDC has been implemented in programmable logic [22,23]. Instead, the challenge is to demonstrate that the proposed architecture behaves robustly under realistic operating conditions and that it integrates naturally into a measurement workflow that can be trusted, repeated, and analyzed.

A key implication of this perspective is that the present work is not framed as a best-case standalone TDC metrology study. Rather, the objective is to characterize a practical timing front-end under experimentally relevant constraints, including internal observability, event trustworthiness, export compatibility, and operating-region robustness. In this context, time-resolved sensing requires not only interpolation but also event qualification and measurement methodology.

The relevance of these issues is also evident in SPAD- and ToF-related systems. State-of-the-art SPAD imagers and photon-timing arrays have shown that timing resolution, histogramming capability, event throughput, dead time, and readout architecture strongly influence the practical quality of the measurement chain [5,24,25,26]. Likewise, in FLIM and TCSPC workflows, dead-time effects, pile-up distortion, and the handling of invalid or biased events are not peripheral nuisances but central parts of the acquisition problem [6,27,28,29]. These observations motivate an architecture in which the event-timing front-end is treated as a measurement subsystem rather than as an isolated interpolation macro. Although the present work does not implement a detector-integrated SPAD system, its architectural choices are motivated by timing-chain requirements that are typical of SPAD-based time-resolved sensing workflows, including asynchronous event arrival, dead-time sensitivity, event trustworthiness, and constrained downstream readout.

The work presented here is therefore positioned as a robust FPGA-based edge-event timing front-end for time-resolved sensing. The implemented system begins with an asynchronous event input and conditions that event for propagation through a carry-chain tapped delay line. The fine state of the delay line is captured coherently, interpreted by cleanup and encoding logic, and combined with a coarse counter to produce a coarse–fine timestamp. In parallel, the system generates event-quality flags that distinguish well-formed events from abnormal or potentially untrustworthy ones. A dead-time-aware handling policy is also included, and accepted events are packetized for export through a lightweight UART interface. The proposed design is thus better understood as a complete event-timing front-end than as a bare carry-chain interpolation block.

The main contribution of the paper is methodological and system-oriented rather than purely interpolation-centric. The proposed front-end combines coarse–fine timestamp formation with explicit event-quality qualification and is characterized through a dual-mode workflow. An internal validation mode, based on ILA observation under MHz-range short-pulse excitation, verifies intrinsic timing-core behavior. An automated export mode, based on UART/MATLAB acquisition, identifies practical host-compatible operating points and separates timing-core capability from constraints imposed by the surrounding measurement infrastructure. In the present work, the front-end implementation and validation are FPGA-based, whereas MATLAB is used only at the host side for data capture, decoding, metric extraction, and figure generation.

The final results support this dual-mode interpretation. Internal ILA observations confirm valid event detection and coherent fine-time extraction under MHz-range short-pulse excitation, while the corrected UART/MATLAB workflow enables quantitative characterization under lower-rate export-compatible conditions. In particular, the baseline condition of 5 kHz and 20 ns yields 1000 captured events per run, essentially a 100% valid-event ratio, negligible saturation, and repeatable exploration of several tens of fine-code bins. A more aggressive condition at 10 kHz and 10 ns retains similarly high event validity while extending the explored fine-code range.

The remainder of the paper is organized as follows. Section 2 positions the contribution with respect to FPGA-based TDCs and sensing-oriented timing systems. Section 3 describes the architecture, implementation strategy, and resource cost of the proposed front-end. Section 4 presents the dual-mode experimental methodology. Section 5 reports the measured results across the main validation and export-characterization experiments. Section 6 discusses the interpretation, limitations, and comparative positioning of the work, and Section 7 concludes the paper.

## 2. Related Work and Quantitative Positioning

The design space of time-to-digital conversion is broad and includes delay-line, Vernier, oscillator-based, multisampling, flash-like, and hybrid approaches [1,2]. Each class occupies a different point in the trade-off space between timing resolution, conversion range, nonlinearity, scalability, power, and implementation complexity. In application-specific integrated circuits, these trade-offs are often addressed through custom analog–digital co-design. In contrast, FPGA-based TDCs must operate within a fixed programmable implementation whose timing properties are only partially controllable, which makes issues such as placement sensitivity, bin nonuniformity, thermometer-code integrity, and implementation-dependent reproducibility especially important [22,23].

Within FPGA implementations, carry-chain-based delay lines have become especially prominent because they reuse native fast-propagation structures and provide a compact route to within-clock-cycle interpolation. Early influential works demonstrated that competitive timing behavior could be obtained by directly exploiting FPGA primitives, thereby establishing the carry-chain tapped delay line (TDL) as a practical interpolation strategy [30,31]. Later work expanded the field toward alternative interpolation resources, higher channel counts, improved calibration, missing-code suppression, lower nonlinearity, and more scalable channel organizations [32,33,34,35,36]. More recent studies continued this trend through multiple-time-coding-line structures, temperature-aware correction, timestamp management, and Artix-7-specific delay-line organization [37,38,39,40].

This literature makes two points especially clear. First, the use of a carry chain by itself does not, on its own, establish a new sensing-oriented contribution. Second, the practical engineering challenges of FPGA-based TDCs remain highly relevant even when the interpolation principle is well known. Reviews of FPGA timing systems emphasize that nonuniform bin widths, bubble errors, missing codes, routing perturbations, calibration overhead, reproducibility across implementations, and dataflow complexity continue to define much of the real difficulty of programmable-logic time interpolation [22,23]. Accordingly, particularly informative contributions are not only those that improve raw timing figures but also those that clarify how timing architectures behave under realistic measurement conditions and system-level constraints.

From the perspective of sensing systems, this broader framing becomes even more important. In ToF imagers, SPAD arrays, and TCSPC/FLIM instrumentation, the timing path cannot be analyzed in isolation from the event-generation and readout chain. Direct ToF sensors, SPAD timing arrays, and histogramming imagers all illustrate that the practical value of a TDC is intertwined with detector architecture, event throughput, dead time, multi-event handling, and output data organization [5,24,25,26]. In FLIM and TCSPC workflows, timing quality is further influenced by pile-up distortion, dead-time effects, and the assumptions used in post-processing to interpret photon arrival data [6,27,28,29]. More broadly, SPAD-oriented literature likewise shows that photon-timing performance must be considered together with detector figures of merit, readout organization, and system-level bandwidth [3,4]. Recent sensor-level work on LiDAR arrays, direct ToF architectures, TDC-sharing SPAD arrays, correlation-assisted pixels, and short-pulse ToF pixel designs further reinforces this application-side perspective [7,8,9,10,11]. In this context, the present work is best understood as a sensing-oriented contribution at the level of the timing front-end and its experimental characterization, rather than as a full detector-integrated sensor demonstration. Table 1 summarizes representative prior work used to position the proposed front-end with respect to FPGA-based TDC implementations and time-resolved sensing systems. Its purpose is not to claim universal superiority, but to provide a compact comparative context for the sensing-oriented positioning adopted in this manuscript.

## 3. Architecture and Implementation

The proposed system is organized as a compact but complete edge-event timing front-end intended for time-resolved sensing experiments in which the most relevant information is encoded primarily in event arrival time. Rather than presenting the design as an isolated delay-line TDC macro, the architecture is conceived as a full measurement-oriented timing path that begins with an asynchronous event input and ends with a validated coarse–fine timestamp together with event-quality metadata suitable for host-side interpretation. This system-level perspective is important because, in practical sensing workflows, the usefulness of a timing front-end depends not only on interpolation capability but also on whether valid events can be distinguished from abnormal ones, whether internal behavior remains observable during experimental development, and whether the resulting timing data can be exported in a form that is trustworthy and analyzable.

**Figure 1:**
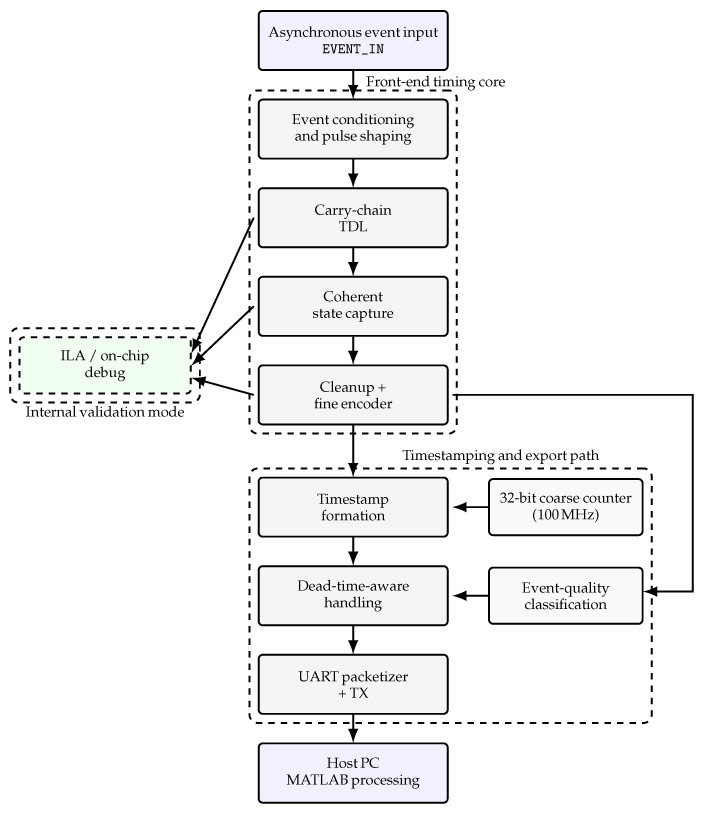
Block diagram of the proposed FPGA-based edge-event timing front-end. The figure emphasizes the vertical signal flow through the front-end timing core, the coarse–fine timestamping and event-quality path, the dead-time-aware export chain, and the separate ILA/debug branch used for internal validation.

Figure 1 summarizes the overall organization of the proposed front-end. The signal flow is represented explicitly from top to bottom, starting from the asynchronous digital event input, denoted in the figure as EVENT_IN, and continuing through the event-conditioning stage, the carry-chain tapped delay line (TDL), the coherent state-capture block, and the cleanup plus fine-encoding stage. The resulting fine-time information is combined with a coarse timing reference in the coarse–fine timestamp formation block, while a parallel event-quality path generates the flags later used for event acceptance and classification. The lower part of the diagram represents the dead-time-aware handling and UART-based export path, which connects the internal timing core to the host-side MATLAB (version R2025a) processing workflow. In parallel, selected internal signals are routed to the ILA/debug branch, highlighting the distinction between the internal validation mode and the automated export mode used later in the measurement campaign.

### 3.1. Event Input Conditioning and Launch into the Fine-Time Path

The entry point of the front-end is an asynchronous digital event input, denoted here as an edge-event signal. In contrast to clock-synchronous logic, this signal is not guaranteed to align with the FPGA system clock, and therefore, its handling must preserve timing information rather than suppress it through immediate synchronization. For that reason, the event is first processed by a dedicated conditioning stage that prepares it for propagation through the fine-time interpolation path.

The role of the conditioning stage is twofold. First, it creates a pulse compatible with the intended TDL observation window. Second, it reduces ambiguity in the launch behavior so that the subsequent delay-line capture remains interpretable. In practical terms, excessively narrow pulses may reduce observability of the propagation front, whereas excessively wide pulses can distort the useful delay-line pattern or increase the likelihood of edge-window saturation. This is also the point at which event handling begins to acquire measurement-level meaning, since distorted or closely spaced events may need to be rejected or explicitly classified rather than accepted blindly.

### 3.2. Carry-Chain Tapped Delay Line

Fine-time interpolation is implemented using the native CARRY4 resources of the Xilinx Artix-7 FPGA. These carry structures provide a fast and relatively regular propagation path that can be repurposed as a tapped delay line, an approach that is well established in FPGA-based TDC design [22,23,30,31]. In the present implementation, the TDL contains 128 effective taps arranged as a vertically constrained carry structure. This organization was selected to reduce placement-induced irregularity and to improve reproducibility across builds.

Although the broader methodological framing of the paper is intended to remain relevant beyond a single vendor, the present realization is specifically a Xilinx Artix-7 implementation and relies on Xilinx-specific resources, most notably CARRY4 delay elements and the integrated logic analyzer (ILA) used for internal validation.

At a conceptual level, the operating principle is straightforward. When the conditioned event arrives, it propagates asynchronously through the carry chain. At the next observation instant, the states of all taps are sampled simultaneously and captured into registers. The resulting sampled pattern provides a spatial snapshot of the edge position within the delay line. In an idealized case, the captured vector resembles a thermometer code, with a transition separating taps already reached by the propagating edge from taps that have not yet been reached. The location of this transition contains the fine-time information.

Given the 128-tap depth used in this implementation and the 100 MHz coarse clock, a nominal average within-clock-cycle granularity on the order of 78 ps would be expected if the full clock interval were sampled uniformly. This value is included only as an approximate scale reference. In an FPGA carry-chain TDL, the effective bin widths are known to be nonuniform and implementation-dependent, so this nominal estimate should not be interpreted as a calibrated resolution figure.

In practice, however, the behavior of a carry-chain TDL inside an FPGA is not ideal. The effective delay associated with each tap may vary because of device-level nonuniformity, routing perturbations, and synthesis or implementation details [34,36,40]. Furthermore, the captured pattern may show local irregularities, especially near the transition region, producing bubble-like artifacts or ambiguous partial transitions. These effects are well known in the literature and help explain why a useful timing front-end must incorporate not only a delay line but also a robust interpretation path.

### 3.3. Coherent State Capture, Cleanup, and Fine-Code Extraction

A central design feature of the proposed system is that the raw delay-line state is captured coherently and preserved long enough to be interpreted in a stable way. Rather than attempting to derive a fine code from loosely observed asynchronous behavior, the system explicitly latches the TDL state and then processes this captured vector through a cleanup and encoding stage. This separation between physical capture and digital interpretation improves robustness and also provides essential debug visibility.

The architecture, therefore, distinguishes between three levels of internal representation:

1. The implicit asynchronous propagation state of the edge inside the carry chain;
2. The latched raw tap vector acquired at the observation instant;
3. The cleaned and encoded fine-time result exported to the rest of the front-end.

This layered interpretation is particularly useful during bring-up and measurement. If an event later appears suspicious at the packet level, the internal latched state can still be inspected to determine if the problem originated in the physical TDL behavior, in the encoding stage, or in a later export block. In this sense, observability is treated as part of robustness rather than as a secondary debug convenience.

The fine-time output is represented with 7 bits in the implementation used for this study, which is consistent with the 128-tap TDL depth. Importantly, the encoded fine result is not exported blindly. It is accompanied by a set of flags that indicate whether the event should be regarded as valid or whether one of several exceptional conditions occurred.

A key role of the cleanup and encoding stage is to transform the coherently captured TDL state into an interpreted event representation that is both exportable and diagnostically meaningful. In practice, this means suppressing non-ideal transition patterns that are not accepted as trustworthy fine-time observations; producing a cleaned, thermometer-like representation for internal interpretation; extracting the encoded fine value when the event is accepted; and generating the companion validity and anomaly indicators used later in the event-quality and dead-time-aware handling stages. This separation between the raw captured state and the interpreted event output is central to the observability-oriented architecture adopted in this work.

In operational terms, the cleanup and encoding flow can be understood as a sequence of six steps. First, the raw tap vector produced by the coherent state-capture block is frozen and made available to the digital interpretation path. Second, the transition structure of that vector is inspected to determine whether it resembles an acceptable monotonic edge pattern or a clearly abnormal case. Third, patterns that are obviously inconsistent with a trustworthy single-edge interpretation are not promoted directly to a valid fine-time event. Fourth, when the captured structure is acceptable, a cleaned thermometer-like representation is derived for internal interpretation. Fifth, the corresponding fine position is encoded into the exported event_fine[6:0] field. Finally, the validity and anomaly indicators are generated so that the downstream event-quality and acceptance logic can distinguish usable events from suspicious ones. This stepwise interpretation is central to the front-end because it separates raw captured TDL behavior from the accepted digital event representation ultimately exposed to the rest of the system.

### 3.4. Coarse Timestamp Path

Because the carry-chain TDL provides only within-clock-cycle information within a limited observation window, it must be paired with a coarse timing reference in order to generate useful timestamps across longer intervals. The proposed architecture therefore includes a 32-bit coarse counter clocked at 100 MHz. This counter provides the clock-cycle-level timing context, while the fine code identifies the event position within the current clock period.

The timestamp produced by the front-end is thus naturally interpreted as a coarse–fine pair. This representation is common in TDC systems because it allows high temporal granularity to be achieved without sacrificing range [1,2]. In the present work, however, the emphasis is not on formal metrological reconstruction of absolute time over a very wide dynamic range but on obtaining robust event timing information under realistic sensing-style operating conditions. For that reason, the most relevant experimental quantities later in the paper are not classical calibrated linearity metrics such as differential nonlinearity (DNL) and integral nonlinearity (INL) but event validity, occupied fine-code range, stability across runs, and the distinction between internal operating capability and export-compatible operating points.

### 3.5. Event-Quality Classification

One of the distinctive aspects of the proposed front-end is the explicit generation of event-quality metadata. Together with the coarse and fine timing fields, the architecture produces a set of flags that classify the sampled event. These flags include a valid-event indication and explicit markers for saturation at the beginning or end of the effective observation window, as well as detection of multi-edge or otherwise abnormal patterns.

This design choice reflects a conservative measurement philosophy. In a laboratory front-end intended for time-resolved sensing, it is often preferable to mark questionable events explicitly rather than silently convert every observed pattern into an apparently valid timestamp. This is especially true when the input excitation is asynchronous, the delay line is finite, and the operating point may vary with stimulus conditions. A front-end that can distinguish usable events from suspicious ones is therefore more valuable for real experiments than one that simply outputs a fine code whenever activity occurs.

The usefulness of such classification is reinforced by the application context. In TCSPC, FLIM, or event-driven ranging systems, even a relatively small fraction of biased or malformed events can distort the interpretation of the timing distribution, especially if those events are not explicitly identifiable [6,27,28]. By exporting event-quality metadata together with each timestamp, the front-end supports cleaner downstream processing and more defensible interpretation of the measured data.

### 3.6. Dead-Time-Aware Event Handling

The architecture also includes a dead-time-aware handling policy. This block is motivated by a practical issue that appears in many event-driven timing systems: when two events arrive too closely spaced in time, the second one may interact with the residual state of the first event or with the transient state of the front-end itself. In such cases, accepting every event indiscriminately may degrade the quality of the dataset or make the resulting timing words difficult to interpret.

To address this, the implementation includes an exclusion mechanism that identifies events arriving within a short hold-off interval after a previously accepted event. Depending on the internal state and the operating mode, such events may be rejected or flagged. In the experimental campaign, the hold-off interval was set to 32 clock cycles, corresponding to approximately 320 ns at 100 MHz, so that accepted events would correspond to interpretable timing states rather than to closely spaced ambiguous captures. The precise role of dead-time-aware handling in this paper is, therefore, not to optimize maximum throughput but to preserve measurement trustworthiness under asynchronous excitation.

From an architectural perspective, this block should therefore be understood as part of the event-acceptance policy of the front-end rather than as a throughput-optimization mechanism. Its role is to prevent the export path and the interpreted timing record from being populated by events that are too closely spaced to be regarded as reliably independent under the internal state assumptions of the measurement chain.

At the behavioral level, the dead-time-aware logic operates as a simple sequential acceptance mechanism. When a qualified event is accepted, the hold-off counter is loaded with the configured exclusion interval of 32 clock cycles. While this counter remains nonzero, subsequent candidate events are prevented from being accepted into the exported event stream, even if activity is observed at the input. Once the counter expires, a new qualified event may again be promoted to the accepted-event path. This behavior is intentionally conservative: rather than attempting to maximize raw event throughput, the block ensures that the interpreted timing record is populated by events that satisfy the front-end’s spacing assumptions and therefore remain easier to analyze and trust.

It is important to interpret this hold-off setting in the context of the experiments reported in this paper. At the UART operating points used in the quantitative campaign ( 5 kHz and 10 kHz), the inter-event spacing is in the 100 μs to 200 μs range, and therefore, the configured 320 ns hold-off does not become a practically limiting factor. In other words, the dead-time-aware mechanism is present and configured during the reported measurements, but it is not strongly exercised by the low-rate laboratory excitation used for the export-compatible campaign. Its relevance would become more apparent in scenarios involving much more closely spaced events, such as detector-driven timing chains or higher-rate asynchronous sensing conditions.

**Figure 2:**
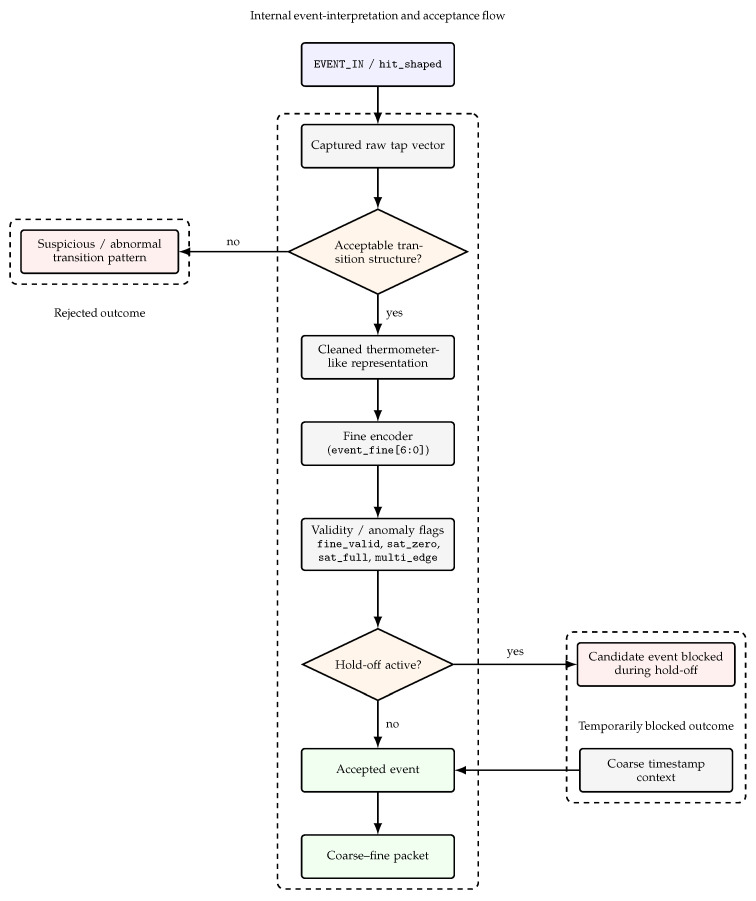
Illustrative internal event-interpretation and acceptance flow of the key processing blocks in the proposed front-end. Unlike the top-level architectural view of Figure 1, the present figure focuses specifically on how captured TDL activity is inspected, classified, and either promoted to an accepted coarse–fine event or diverted to a suspicious or blocked candidate-event outcome.

While Figure 1 provides the top-level architectural organization of the front-end, Figure 2 focuses specifically on the internal interpretation and acceptance flow of the key event-processing blocks. Its purpose is not to repeat the system-level architecture but to clarify how captured TDL activity is inspected, classified, and either promoted to an accepted coarse–fine event or diverted to a suspicious or blocked candidate-event outcome. Table 2 complements this view by summarizing the main inputs, outputs, and functional role of the principal processing blocks.

### 3.7. UART Packetization and Streaming

Accepted events are exported through a lightweight UART streamer, providing a simple and transparent path suitable for laboratory-scale acquisition and MATLAB-based analysis. This export link is not intended to maximize throughput; its methodological role is to define the practical host-visible regime used for repeatable automated characterization. Crucially, the maximum rate at which events can be exported through this link is not the same as the maximum rate at which the internal front-end can detect and classify events—an asymmetry that directly motivates the dual-mode strategy described in Section 4.

### 3.8. Implementation Platform and Physical Constraints

The complete system is implemented on the Digilent Basys 3 platform, based on a Xilinx Artix-7 FPGA. The choice of this platform is deliberate: it is low cost, widely available, and well-suited for experimental work in which accessibility and reproducibility matter. The system clock is 100 MHz, and the UART interface operates at 921,600 baud.

Because carry-chain timing behavior is sensitive to physical organization inside the FPGA design, placement control plays an important role in the implementation. The TDL is therefore constrained to a consecutive vertical carry structure in order to reduce variability associated with uncontrolled place-and-route solutions. This does not make the structure perfectly uniform, but it helps improve consistency and aligns with established good practice in FPGA-based timing design [22,23].

### 3.9. Internal Observability and Representative Waveform Behavior

A further strength of the implementation is that internal observability was treated as a first-class design goal. In addition to the packetized UART stream, the front-end exposes internal states and event-quality signals to the integrated logic analyzer. This includes the cleaned thermometer representation, the encoded fine-time result, the event strobe, and the main validity and saturation flags. Such visibility greatly simplifies debugging and was essential in distinguishing between problems intrinsic to the timing core and problems caused by the export path.

**Figure 3:**
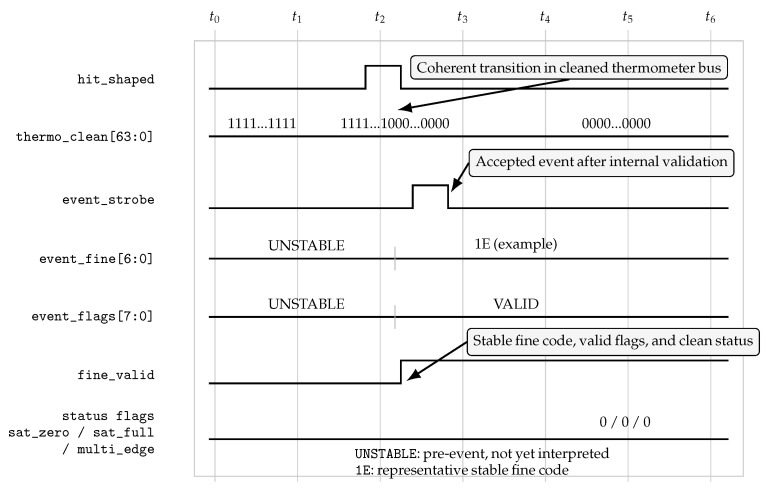
Didactic waveform diagram illustrating the signal-level interpretation of a valid internal event in the validation mode. The signal names, timing relationships, and flag states shown are representative of the coherent behavior observed in the processed ILA captures described in Section 5.2 and quantified in Figure 4. The label UNSTABLE denotes pre-stabilization or non-interpreted values before event acceptance, whereas 1E is a representative example of a stable encoded fine value after acceptance. Likewise, the transition from a pre-event flag word to VALID indicates the assertion of a stable accepted-event classification. The diagram is presented in schematic form to make the temporal sequencing between blocks explicit; the quantitative evidence for this behavior is provided by the ILA CSV analysis summarized in Figure 4.

**Figure 4:**
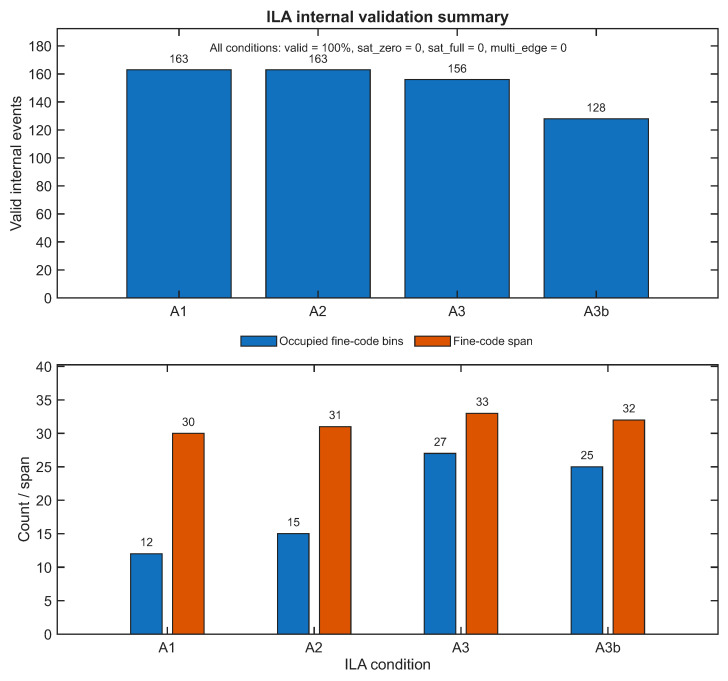
Summary of the main ILA-based internal validation results for conditions A1, A2, A3, and A3b. The upper panel reports the number of valid internal events observed in each capture window, while the lower panel shows the occupied fine-code bins and the fine-code span. All these conditions yielded valid internal events without zero-end saturation, full-end saturation, or multi-edge ambiguity.

Figure 3 provides a didactic waveform diagram illustrating the signal-level interpretation of a valid internal event in the validation mode. The diagram is schematic rather than a direct screen capture, and its purpose is to clarify the temporal sequencing between the internal shaped hit (hit_shaped), the cleaned thermometer representation (thermo_clean[63:0]), the accepted-event strobe (event_strobe), the encoded fine output (event_fine[6:0]), the event-quality word (event_flags[7:0]), the explicit fine-valid indication (fine_valid), and the main status indicators (sat_zero, sat_full, and multi_edge). In the pre-event region, the labels UNSTABLE denote values that are not yet interpreted as stable event outputs. After the accepted-event strobe, the fine output settles to the representative code 1E, used here only as an illustrative example of a stable encoded value rather than as a special or preferred code. Likewise, the transition from UNSTABLE to VALID in event_flags[7:0] indicates the transition from a pre-event/non-interpreted flag word to a stable accepted-event classification. The assertion of fine_valid and the clean 0/0/0 status pattern indicate that the shown event is accepted without zero-end saturation, full-end saturation, or multi-edge ambiguity. The quantitative behavioral evidence for this type of coherent internal behavior is provided separately in Figure 4.

Together, Figure 1 and Figure 3 provide a compact visual summary of how an asynchronous event is processed through the front-end and represented as a validated coarse–fine timing word with associated status information.

### 3.10. Implementation Cost and Resource Utilization

Table 3 summarizes the implementation cost on the target Basys 3/Artix-7 platform. A distinction is made between the debug-enabled build used during ILA-based internal validation and the practical functional design itself. This distinction is important because the integrated ILA core and associated debug hub dominate the resource usage of the instrumented implementation, particularly in LUTs, registers, and block RAM. By contrast, the sensing front-end remains comparatively lightweight. Based on the reported post-implementation hierarchical utilization, the functional design is estimated to require around 1.3 k LUTs, about 0.5 k registers, 381 occupied slices, no block RAM, and only one global clock buffer. These figures support the interpretation of the proposed architecture as a compact timing front-end whose large debug-enabled footprint is driven primarily by observability requirements rather than by the timing architecture itself.

## 4. Experimental Methodology

### 4.1. Methodological Rationale

A central premise of this work is that the behavior of a sensing-oriented timing front-end cannot be assessed adequately through a single experimental regime. During experimental development, it became clear that two distinct but complementary issues had to be addressed. First, the intrinsic internal behavior of the FPGA timing core had to be validated under short-pulse, relatively high-rate excitation conditions representative of an edge-driven sensing front-end. Second, the practical ability to export, decode, and analyze large sets of timestamped events had to be evaluated under host-compatible operating conditions. These two requirements are not equivalent, and in the present system, they are not governed by the same limiting mechanism.

This observation led to the definition of a dual-mode methodology composed of an internal validation mode and an automated export mode. The first mode is centered on integrated logic analyzer (ILA) observation of internal signals and is intended to verify that the timing core itself behaves coherently under MHz-range short-pulse excitation. The second mode is centered on UART streaming and MATLAB-based post-processing and is intended to support repeatable large-set quantitative analysis under export-compatible operating points. The methodology is therefore stimulus-aware in two complementary senses: it considers both the effect of input conditions on the internal TDL behavior and the practical constraints imposed by the communication path used for automated host-side logging.

This separation is particularly important in the present work because the experimental campaign was carried out with the Digilent Digital Discovery generator integrated into the laboratory setup, and the accessible stimulus space was therefore defined by its timing configuration granularity. In practice, this meant that event frequency and minimum achievable pulse width were not fully independent, since the available duty-cycle resolution coupled both quantities. Rather than treating this constraint as incidental, the methodology was defined explicitly around it so that the reported operating regions would remain reproducible within the actual laboratory workflow used to validate the front-end.

Likewise, the UART output path introduces a practical ceiling on the rate at which timestamped events can be exported and processed reliably. If these external constraints were ignored, the measured operating region of the complete system could easily be misinterpreted as an intrinsic limitation of the TDL timing core itself. The methodology adopted here is designed precisely to avoid that conceptual error.

It is also important to clarify the intended scope of the measurements. The goal of the experimental campaign is not to provide a full TDC metrology study in the classical sense, with exhaustive calibrated DNL/INL, PVT dependence, or best-case single-shot precision benchmarking. Instead, the purpose is to characterize the front-end as an experimentally usable timing subsystem under realistic sensing-style constraints, with emphasis on event validity, operating-region robustness, internal observability, and export-compatible behavior.

### 4.2. Measurement Objectives

The experimental campaign was structured around six specific methodological objectives:

1. To verify valid event detection and classification by the internal timing front-end under MHz-range excitation with short pulses.
2. To identify a safe operating point for reliable large-set automated export through UART.
3. To assess the repeatability of that safe export operating point across multiple runs and acquisition sessions.
4. To evaluate front-end robustness against pulse-width variation at a fixed event rate.
5. To determine whether a more aggressive operating point can preserve high validity while extending the explored fine-code range.
6. To characterize how the transition from the safe to the aggressive export regime evolves as frequency is increased.

The first objective is addressed through the ILA-based internal validation mode. Objectives two through six are addressed through the UART/MATLAB export mode. This decomposition ensures that each experiment addresses a well-defined methodological objective and prevents the measurement campaign from degenerating into a mere catalog of operating conditions.

### 4.3. Experimental Modes

#### 4.3.1. Internal Validation Mode

In the internal validation mode, the objective is not to export thousands of events to the host PC but to verify the behavior of the timing core itself. This mode uses the integrated logic analyzer to capture internal signals such as the cleaned thermometer representation, the encoded fine-time result, the event strobe, and the main event-quality flags. The main experimental conditions in this mode are in the MHz range, with short pulses close to the regime that had previously shown coherent internal TDL behavior during bring-up.

Representative conditions in this mode include approximately 1.00 MHz and 1.01 MHz with pulse widths close to 10 ns. These conditions are not intended to define the final automated operating point of the complete system. Instead, they are used to demonstrate that the internal front-end can detect valid events, produce coherent thermometer-like patterns, and extract stable fine codes in a regime closer to the temporal dynamics expected from an event-driven sensing front-end.

This mode is therefore especially valuable for separating internal timing capability from export bandwidth. A valid internal event observed through ILA does not imply that the UART link can sustain continuous high-rate export of all such events. What it does show is that the TDL-based timing core itself is not intrinsically limited to the much lower event rates later used for automated host logging.

#### 4.3.2. Automated Export Mode

In the automated export mode, the purpose shifts from qualitative internal validation to quantitative large-set characterization. In this regime, each accepted event is packetized and exported through the UART link to a MATLAB acquisition and analysis pipeline. This mode is used to collect repeatable statistics on large event sets and to generate the summary metrics that later structure the results section, including valid-event ratio, saturation rate, multi-edge occurrence, occupied fine-code bins, and explored fine-code range.

Unlike the internal validation mode, the export mode must satisfy both internal timing quality and practical host-side communication constraints. An operating point is therefore considered useful only if it simultaneously preserves a high ratio of valid events, avoids strong saturation or multi-edge behavior, allows reliable packet export and decoding, and yields a sufficiently rich fine-code distribution to support meaningful analysis.

The export mode is thus deliberately more conservative than the ILA mode, not because the timing core ceases to function at higher rates, but because the end-to-end measurement chain must remain reliable, repeatable, and interpretable.

### 4.4. Stimulus Constraints and Operating-Point Selection

The selection of operating points was strongly influenced by the practical limitations of the external signal generator. In the Digilent Digital Discovery setup used in this work, the minimum duty cycle that could be configured reliably was 0.01%. As a result, the minimum pulse width depends on the selected event frequency. For example, at 1 kHz, the minimum available pulse width becomes approximately 100 ns, whereas at 5 kHz, the same minimum duty corresponds to approximately 20 ns, and at 10 kHz, it corresponds to approximately 10 ns. This coupling is central to understanding the final experimental matrix.

From internal exploratory observations, it was found that short pulses around 10 ns produced the most convincing internal TDL behavior in the ILA, whereas substantially wider pulses were not needed for internal validation at MHz-range excitation. However, a fully automated campaign at an MHz-rate event injection would not be compatible with reliable export through the UART link. A compromise, therefore, had to be found for the quantitative campaign.

The selected baseline export condition was 5 kHz with a pulse width of 20 ns, corresponding to the minimum available duty at that frequency. This operating point was prioritized because it balances UART bandwidth constraints with the temporal dynamics of the internal timing core while remaining close to the experimentally preferred short-pulse regime. A second, more aggressive export condition was selected at 10 kHz with a pulse width of 10 ns. For brevity, this more exploratory high-rate condition is referred to hereafter as the aggressive operating point. The comparison between these two conditions becomes one of the central quantitative results of the paper.

### 4.5. Experimental Matrix

The final experimental matrix is organized into six blocks.

Block A: Internal ILA validation.

This block includes representative internal captures in the MHz range, such as approximately 1.01 MHz/1.01% and 1.00 MHz/1.00%. Its purpose is to demonstrate valid internal event detection, coherent thermometer behavior, and clean event flags under short-pulse excitation. These experiments provide the main evidence that the internal TDL front-end is not fundamentally limited by the much lower event rates used later in export mode.

Block B: Baseline export characterization.

This block consists of repeated runs at 5 kHz and 20 ns. Its purpose is to establish the safe export operating point. Metrics extracted from this block include the number of captured packets, the valid-event ratio, the saturation percentages, the multi-edge rate, the minimum and maximum observed fine code, and the number of occupied fine-code bins.

Block C: Pulse-width robustness.

This block keeps the frequency fixed at 5 kHz while varying the pulse width across 20 ns, 100 ns, and 200 ns. Its purpose is not necessarily to identify a catastrophic failure boundary but to determine if the baseline remains robust under moderate widening of the input pulse within the practically accessible range.

Block D: Safe versus aggressive export comparison.

This block compares the safe baseline of 5 kHz/20 ns against the more aggressive point of 10 kHz/10 ns. It is intended to assess if the front-end can maintain high event validity while extending the explored fine-code range under a more demanding export condition.

Block E: Intermediate export-frequency sweep.

This block bridges the gap between the conservative export regime and the more demanding short-pulse operating point. In this experiment, the pulse width is kept fixed in the short-pulse regime while the event frequency is stepped progressively between the safe and aggressive conditions. Its purpose is not to identify a single abrupt export limit but to determine if the export-visible degradation emerges gradually or abruptly as the system is pushed toward higher-rate operation. For consistency, the same metrics extracted throughout the UART campaign are used here as well, including valid-event ratio, saturation indicators, occupied fine-code bins, and fine-code span.

Block F: Repeatability and temporal stability.

This block includes additional repetitions of the baseline condition and repeated acquisitions performed later in time. Its purpose is to verify that the selected baseline is not merely a one-off favorable condition but a reproducible and temporally stable operating point.

Taken together, the matrix defines a coherent experimental progression: internal validation, baseline establishment, robustness assessment, safe-versus-aggressive comparison, and intermediate operating-space exploration. Table 4 summarizes the resulting experimental matrix, including the two operating modes, the main block-level objectives, the representative conditions, and the principal metrics used throughout the measurement campaign.

### 4.6. UART Packet Integrity and Host-Side Processing

The automated export mode relies on a custom UART streamer that packetizes each accepted event into a compact frame containing a header, coarse timestamp information, fine code, event flags, and checksum. The relevance of this export path is that it defines the practical host-visible regime used for repeatable automated characterization.

During experimental development, a defect was identified in the byte-sequencing logic of the initial streamer implementation, producing packet misalignment in early runs. After correcting this issue, the UART path became stable enough to support the quantitative campaign reported in the paper. Only post-fix runs were retained for final analysis, so the reported results correspond to a validated end-to-end export chain.

On the host side, MATLAB was used only for packet capture, decoding, metric extraction, experiment logging, and figure generation. The timing front-end itself was implemented and experimentally validated on an Xilinx Artix-7 FPGA (Basys 3 platform), while the MATLAB processing flow reads the UART stream, reconstructs event fields, decodes the flags, computes per-run summaries, and updates the experiment workbook. In parallel, separate scripts were developed for the ILA CSV workflow, allowing internal captures to be processed into event-level summaries and supporting the unified use of ILA and UART evidence in the final paper.

### 4.7. Recorded Metrics

The methodology emphasizes metrics that are directly meaningful for sensing-oriented front-end operation. For each export run, the following quantities were recorded:

- Number of accepted packets;
- Valid-event percentage;
- Saturation-at-zero percentage;
- Saturation-at-full percentage;
- Multi-edge percentage;
- Minimum observed fine code;
- Maximum observed fine code;
- Number of occupied fine-code bins.

These metrics were selected because they jointly characterize whether the front-end is merely active or actually useful. A good operating point should not only yield packets but also do so with high event validity, low abnormal-event rates, and a sufficiently rich fine-code distribution. The number of occupied bins and the explored fine-code range are especially relevant in this work because they provide an experimentally accessible measure of whether the front-end is exploring a useful portion of its internal timing state space. In this paper, the reported fine-code span is used as an empirical descriptor of the explored code interval within the corresponding processed dataset, rather than as a claim of calibrated linear code-space coverage. More specifically, the span is defined as the difference between the maximum and minimum fine-code values observed within a given acquisition run or aggregated dataset, expressed in units of fine-code bins. Since the encoder output is a 7-bit word and the TDL contains 128 taps, valid fine codes nominally range from 0 to 127. However, the reported span values in some experiments exceed this nominal range because the span metric is computed across the aggregated dataset, combining multiple runs, each of which may explore a different sub-interval of the fine-code axis depending on the exact phase relationship between the asynchronous input event and the system clock at the moment of capture. The span, therefore, reflects the union of fine-code intervals explored across all contributing acquisitions, not a single-run excursion that violates the 7-bit constraint. Individual per-run fine-code values remain within the 0–127 range enforced by the encoder.

For the ILA mode, the metrics are necessarily more structured and less purely statistical because the capture depth and trigger conditions differ from the UART export regime. Even so, the ILA CSV processing allows one to count event strobes, valid internal events, saturation and multi-edge occurrences, and the occupied fine-code range for the internal validation conditions. These data are not used as a substitute for the UART quantitative campaign but as complementary evidence that the internal front-end behaves coherently under higher-rate excitation.

### 4.8. Criteria for Experimental Success

The experimental campaign was not judged by a single best-case timing number. Instead, each block had its own criterion of success.

For the ILA validation mode, success required the observation of coherent, cleaned thermometer patterns; stable fine-code extraction; valid-event flags; and the absence of clear invalid conditions in representative events. For the baseline UART mode, success required reliable capture of 1000 events per run together with a near-unity valid-event ratio and low saturation. For the width sweep, success meant determining if the baseline behavior remained stable as pulse width increased. For the safe versus aggressive comparison, success meant identifying if a more demanding operating point could preserve high validity while expanding the explored fine-code range. For the intermediate sweep, success meant determining if the transition between conservative and aggressive export regimes remained gradual and interpretable. Finally, for the repeatability block, success required that the baseline metrics remain consistent across multiple runs and across measurements separated in time.

This way of defining success is particularly appropriate for a sensing-oriented front-end. Rather than maximizing a single isolated implementation metric, the methodology seeks to identify operating points that are simultaneously meaningful, trustworthy, and experimentally reproducible.

## 5. Experimental Results

### 5.1. Overview of the Experimental Evidence

The experimental results are presented according to the dual-mode methodology introduced in Section 4. Internal ILA-based evidence is first used to verify that the timing front-end behaves coherently under MHz-range short-pulse excitation, whereas the UART/MATLAB campaign is then used to identify and characterize operating points that are meaningful from the viewpoint of practical, reproducible, large-set measurements. This organization avoids conflating the intrinsic internal capability of the TDL-based timing core with the more restrictive operating region of the complete host-visible export chain.

### 5.2. Internal Validation Under MHz-Range Excitation

The first set of results concerns the internal validation mode. In this regime, the purpose is not to maximize host-visible export throughput but to verify that the timing front-end itself behaves coherently under short-pulse excitation in the MHz range. Figure 3, described above, provided a stylized ILA-like waveform diagram illustrating the expected internal behavior of a valid event in this mode. In this figure, the UNSTABLE annotations denote pre-stabilization or non-interpreted values, whereas 1E and VALID denote representative stable outputs after event acceptance. The internal shaped hit is followed by a coherent transition in the cleaned thermometer bus, an accepted-event strobe, a stable fine-code output, and clean event-quality indicators without saturation or multi-edge ambiguity.

For clarity, the representative ILA conditions labeled A1, A2, A3, and A3b all use 10 ns pulses and differ only slightly in excitation frequency, spanning approximately 1.00 MHz to 1.01 MHz. In particular, A1 and A3b correspond to the slightly detuned 1.01 MHz case, whereas A2 and A3 correspond to the 1.00 MHz case.

Figure 4 summarizes the quantitative results obtained from the processed ILA captures for the main internal-validation conditions A1, A2, A3, and A3b. Since all accepted internal events in these captures were classified as valid, and no saturation or multi-edge pathologies were observed, the figure emphasizes two more informative descriptors: the number of valid internal events observed within the acquisition window and the extent of the occupied fine-code space. In this way, the figure complements the waveform-level interpretation of Figure 3 with compact quantitative evidence that the internal timing front-end remains coherently active under the selected MHz-range short-pulse conditions. The absolute number of valid internal events observed in each ILA capture should be interpreted as a descriptor of activity within the corresponding capture window rather than as a normalized throughput metric, since the ILA observation context is determined by trigger and windowing conditions rather than by a long statistical acquisition protocol.

The results in Figure 4 show that the internal validation mode is not limited to isolated anecdotal observations. Instead, the processed ILA consistently exhibits valid internal activity together with nontrivial fine-code occupation across all selected conditions. The number of valid events observed in the capture window remains substantial in every case, while the occupied-bin and fine-span metrics confirm that the internal front-end is exploring a meaningful portion of the fine-time state space.

This result should be interpreted carefully. It does not imply that the complete host-visible acquisition chain can sustain continuous MHz-range export. Rather, it shows that the internal front-end remains coherent under conditions that are more demanding than those adopted for the automated UART campaign. In this sense, the ILA evidence supports the view that the export-compatible operating points are constrained primarily by the full measurement chain rather than by an intrinsic inability of the timing core to operate under higher-rate short-pulse excitation.

The reported MHz-range ILA validation should therefore be interpreted as representative internal validation under the stimulus and capture conditions available in the present study, rather than as an absolute ceiling of the timing core itself. Extending this validation toward higher sustained rates would require a correspondingly faster stimulus/capture chain and is therefore left for future work.

### 5.3. Safe Export Baseline: Reproducibility and Stability

The central quantitative result of the paper is the identification of a safe export operating point at 5 kHz and 20 ns. This condition was selected because it preserves a short pulse width compatible with the useful internal regime while remaining safely within the bandwidth envelope of the corrected UART/MATLAB acquisition chain. The first question addressed experimentally was whether this operating point is merely functional or if it is also repeatable and stable.

**Figure 5:**
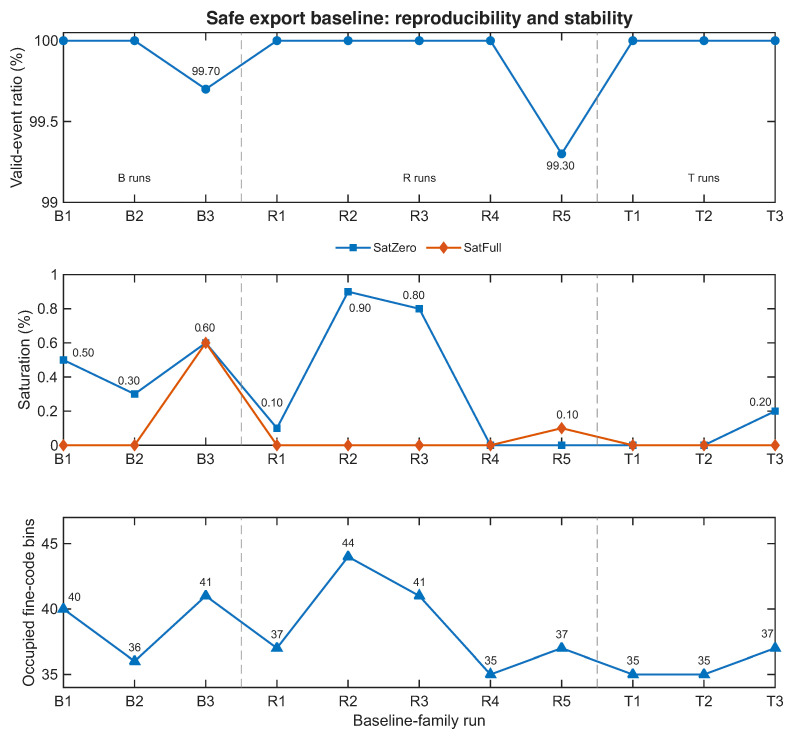
Repeatability of the safe export baseline across the initial, extended-repeatability, and temporal-stability families. The figure reports the evolution of the valid-event ratio, saturation indicators, and occupied fine-code bins over repeated runs.

To answer that question, the baseline condition was evaluated across three initial runs (B1–B3), five additional repeatability runs (R1–R5), and three later-session temporal-stability runs (T1–T3). The resulting aggregate statistics are summarized in Table 5, while Figure 5 shows the run-to-run evolution of the key baseline metrics.

Several important observations follow from these results. First, the packet-based acquisition chain remained fully usable throughout the baseline family, with 1000 accepted packets obtained in every run. This confirms that the corrected UART path was not merely functional in isolated tests but stable enough to sustain repeated acquisition across the full baseline block.

Second, the valid-event ratio remained extremely high across all baseline-family runs. While individual runs showed small variations, the aggregate mean of 99.92% indicates that the operating point is not only usable but also highly selective in accepting events that remain interpretable according to the front-end’s own quality criteria. This is an important result in the context of sensing-oriented timing front-ends, because it means that the exported timestamp stream is dominated by events that the system itself considers trustworthy. The run-to-run standard deviation of the valid-event ratio across the 12-run baseline family was 0.21 percentage points, confirming that the operating point is not only high-performing in isolation but also statistically stable across repeated acquisitions.

Third, the saturation metrics remained very low. Saturation at the beginning of the effective observation window stayed at the sub-percent level on average, while saturation at the full end was even smaller. Multi-edge behavior was negligible. These results suggest that the chosen baseline successfully balances a sufficiently short pulse width with a sufficiently conservative export rate, thereby avoiding invalid operation without collapsing the explored timing region.

Finally, the number of occupied fine-code bins remained consistently in the high-30s to low-40s range. This complements the validity statistics. A front-end that yields nearly 100% valid events but collapses into only one or two fine codes would not be very useful. In contrast, the present results show that the baseline operating point combines high validity with nontrivial fine-code exploration. Across the 12 baseline-family runs, the corresponding run-to-run variations remained modest, with no indication of systematic drift or collapse of the selected operating point.

### 5.4. Fine-Code Distribution Under the Baseline Condition

**Figure 6:**
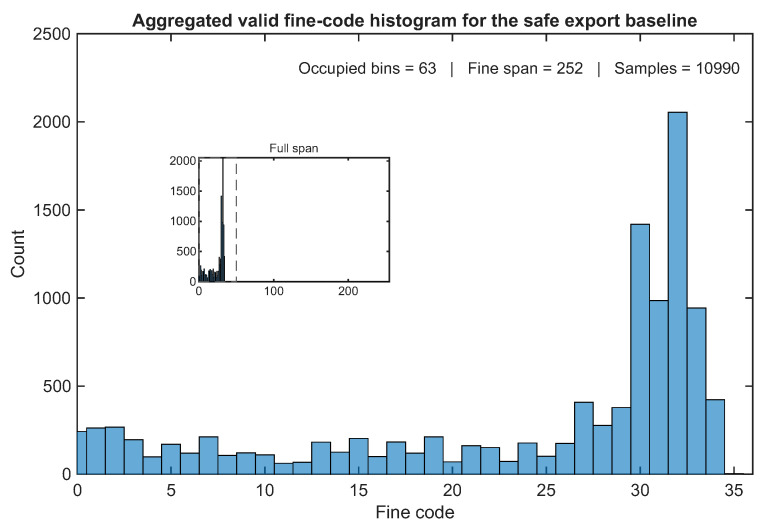
Aggregated histogram of valid fine codes for the safe export baseline family. The main panel zooms into the lower-code region where most valid fine-code samples are concentrated, while the inset preserves the full explored fine-code span. The histogram is formed by combining all valid fine-code samples from the baseline, repeatability, and temporal-stability runs. The resulting distribution confirms that the baseline operating point remains highly valid while occupying a nontrivial portion of the internal fine-code space.

The aggregate fine-code behavior of the baseline family is illustrated in Figure 6, which reports the histogram obtained by accumulating all valid fine-code samples from runs B1–B3, R1–R5, and T1–T3. The purpose of this figure is not to extract formal metrological quantities such as DNL or INL but to verify that the safe export baseline remains associated with a nontrivial distribution of valid fine codes rather than collapsing into a narrowly confined subset of the fine-time space. In this sense, the histogram complements the repeatability trends of Figure 5 by showing how the valid-event stream is distributed internally across the fine-code axis.

To improve the readability of this distribution, the main panel of Figure 6 focuses on the lower-code region where most valid fine-code samples are concentrated, whereas the inset preserves the full explored fine-code span. Although the occupied span extends to substantially higher sparse codes, most valid samples cluster in the lower-code region shown in the main panel. This presentation avoids visually diluting the dominant occupancy structure while still making the broader explored range explicit.

In this context, the reported fine-code span should be interpreted strictly as a descriptor of the numerical interval covered by the processed valid-event population within the corresponding analysis dataset. It is not intended to represent the direct calibrated range of the exported event_fine[6:0] field nor to imply that the 7-bit fine word alone linearly spans that full numerical extent. Rather, the span metric is used here only to summarize how broadly the processed valid-event distribution extends within the analysis space adopted for comparative occupancy interpretation.

Figure 6 shows that, even though most valid samples are concentrated in a lower-code region, the baseline front-end behavior cannot be reduced to a single preferred code. Instead, valid fine codes are distributed over a broad interval, consistent with the occupied-bin counts reported in Figure 5 and Table 5. This observation is important because it rules out a trivial interpretation of the high valid-event ratio. In other words, the excellent event validity of the baseline is not obtained by collapsing the front-end into only one or two recurrent codes but by operating in a regime that remains both selective and internally informative. The sparse higher-code occurrences visible in the inset are retained to preserve the full explored range of the processed valid-event dataset. In the absence of a dedicated calibration-oriented interpretation of that extended region, they are not used here as the basis for a separate physical claim, but simply as part of the broader occupancy picture associated with the baseline regime.

At the same time, the aggregated histogram is not uniform, and this point deserves explicit discussion. In an FPGA carry-chain TDL, nonuniform bin widths and implementation-dependent delay perturbations are well known, so the observed distribution should not be interpreted as evidence of ideal linearity. The present figure is therefore used as a qualitative indicator of nontrivial fine-code occupation rather than as a substitute for formal DNL/INL analysis. The nonuniformity observed here is compatible with the expected behavior of an uncalibrated FPGA TDL and does not invalidate the main conclusion of the figure, namely that the baseline operating point preserves access to a meaningful portion of the internal fine-time space.

Although the histogram of Figure 6 is useful as an occupancy descriptor of the safe export regime, it is not in itself a formal code-density metrology result. For that reason, it is useful to distinguish it from the type of preliminary DNL/INL and RMS evidence that would be considered suitable for dedicated TDC-oriented reporting.

### 5.5. Preliminary Code-Density and Fine-Time Dispersion Characterization

To complement the main operating-region analysis, a supplementary metrological characterization was performed in order to obtain a first-order view of the uncalibrated temporal behavior of the FPGA carry-chain front-end. The metrological indicators reported in this subsection were extracted from a dedicated filtered UART campaign assembled specifically for code-density and local-dispersion analysis and, therefore, use a substantially larger sample count than the baseline occupancy histogram reported earlier. In the present case, the DNL/INL/RMS estimates are based on approximately 120,000 filtered samples collected under a dedicated metrological acquisition at 5 kHz event frequency and 20 ns pulse width—the same stimulus condition as the safe export baseline (but with a substantially longer acquisition duration selected specifically to accumulate sufficient counts for code-density evaluation). This acquisition is therefore stimulus-compatible with the baseline family but constitutes a separate, longer-duration dataset assembled for metrological purposes. Figure 6, by contrast, is based on the aggregated baseline-family runs (B1–B3, R1–R5, T1–T3) and serves a different interpretive purpose.

**Figure 7:**
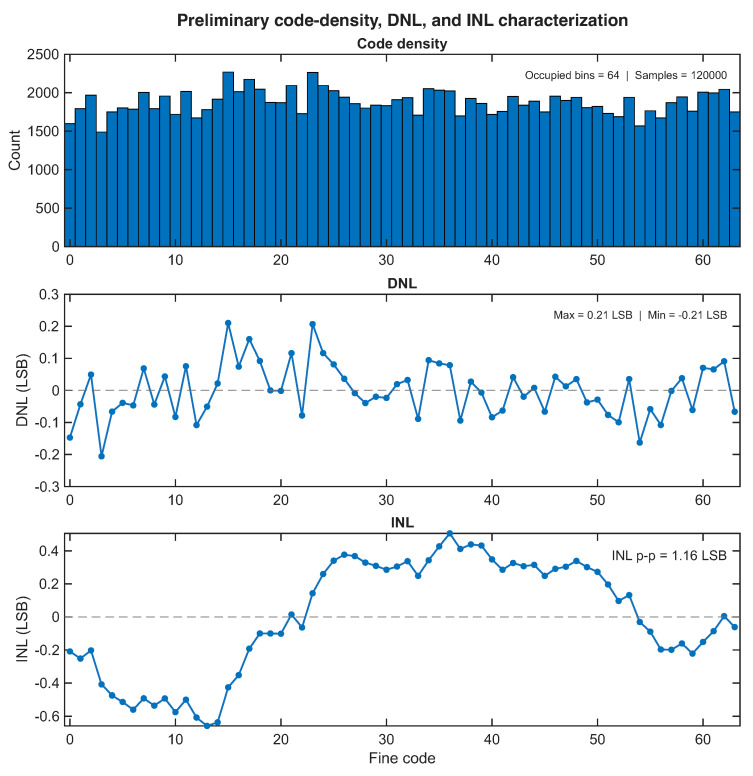
Preliminary code-density characterization of the filtered fine-code output. The upper panel reports the measured fine-code occupancy histogram, while the middle and lower panels show the corresponding DNL and INL estimates extracted from valid UART-exported events under asynchronous excitation.

Figure 7 reports the resulting preliminary code-density characterization obtained from filtered UART captures under asynchronous excitation. The upper panel shows the fine-code occupancy histogram, while the middle and lower panels report the corresponding DNL and INL estimates. The fine-code range covered in this preliminary metrological analysis is narrower than the one visible in the aggregated baseline histogram of Figure 6. This difference is expected because the two figures serve different interpretive purposes: Figure 6 is an occupancy-oriented descriptor of the safe export regime over the full explored fine-code span, whereas the present analysis focuses on the metrologically useful region selected from dedicated filtered captures for code-density evaluation. As expected for an uncalibrated FPGA TDL, the code-density distribution is not perfectly uniform, which is consistent with the known nonuniformity of carry-chain bin widths and implementation-dependent delay perturbations. The DNL oscillates around zero with moderate excursions, whereas the INL evolves smoothly over the analyzed fine-code region without catastrophic discontinuities. These results should, therefore, be interpreted as a preliminary descriptor of bin nonuniformity rather than as a substitute for a dedicated calibration-grade linearity campaign. The dedicated metrological acquisition should, therefore, be interpreted as a supplementary characterization dataset rather than as part of the baseline-family operating-envelope experiments defined in Section 4.

**Figure 8:**
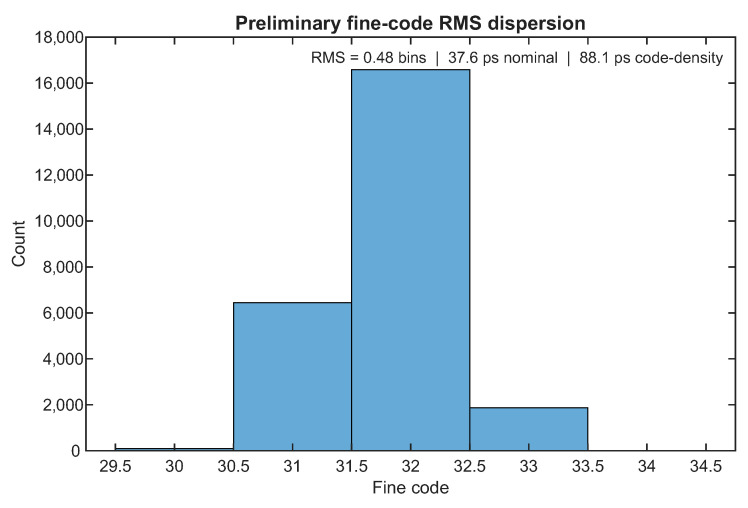
Preliminary fine-code RMS dispersion obtained under repeatability-oriented conditions. The histogram is localized around a relatively narrow subset of fine codes, supporting its interpretation as a local dispersion metric rather than as the global spread associated with asynchronous exploration of the fine-code space.

Figure 8 reports the corresponding preliminary fine-code RMS dispersion result. In this case, the relevant visual signature is a localized distribution concentrated around a relatively narrow subset of codes, which is consistent with a repeatability-oriented interpretation of RMS under approximately fixed timing conditions. This should be distinguished from the much broader histograms used elsewhere in the paper to illustrate global fine-code occupancy under asynchronous exploration of the operating space. In the present context, the RMS quantity is used only as a compact indicator of local fine-time dispersion and should not be interpreted as a formal single-shot precision benchmark. The difference between the nominal-bin and code-density-derived RMS estimates reflects the nonuniformity of the underlying FPGA TDL. The nominal estimate assumes a uniform average bin width, whereas the code-density-derived estimate incorporates the measured unequal occupation of the fine bins and is therefore more conservative. These two values should not be interpreted as contradictory but as two complementary ways of expressing the same local dispersion result under different assumptions about bin uniformity.

Table 6 summarizes the corresponding preliminary metrological indicators. These quantities provide a compact first-order view of the uncalibrated temporal behavior of the front-end. In particular, the occupied-bin count and sample count quantify the extent of the analyzed region, the DNL and INL values provide a preliminary description of bin nonuniformity, and the RMS quantities summarize the local fine-time dispersion in both code units and approximate temporal units.

Taken together, these preliminary results provide a useful metrological complement to the main sensing-oriented characterization presented in the paper. They indicate that the front-end exhibits the expected bin nonuniformity of an uncalibrated FPGA TDL while still supporting an experimentally interpretable fine-time response over a nontrivial portion of the accessible code space. At the same time, the scope of these results remains deliberately limited: they should be understood as first-order descriptors of uncalibrated behavior, not as a replacement for a full swept-delay, calibration-heavy TDC metrology campaign. Their role in the paper is therefore supplementary rather than central: they provide a first-order metrological context for the front-end without altering the main operating-region narrative established by the baseline, robustness, and operating-envelope experiments.

### 5.6. Robustness Against Pulse-Width Variation at Fixed Frequency

**Figure 9:**
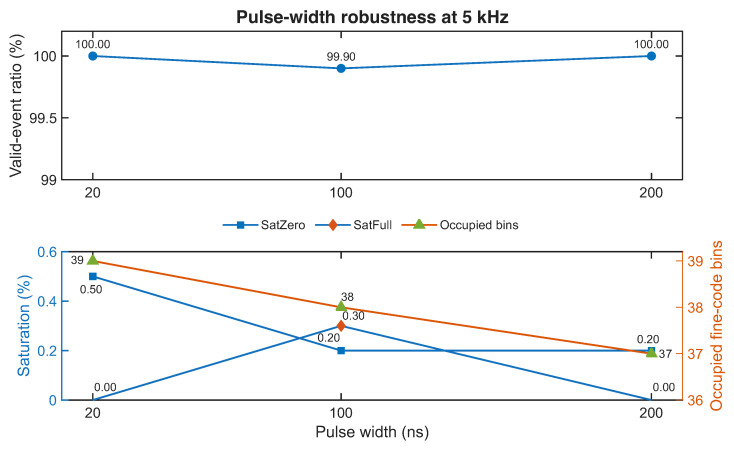
Robustness of the front-end against pulse-width variation at 5 kHz. The upper panel reports the valid-event ratio, which remains essentially unchanged across the explored pulse-width range. The lower panel combines the saturation indicators (SatZero and SatFull, left axis) with the number of occupied fine-code bins (right axis), showing that moderate pulse-width widening produces only small changes in export-visible behavior.

The next question addressed experimentally was whether the baseline behavior remains robust when the pulse width is increased while the frequency is held fixed at 5 kHz. This was explored through three conditions: 20 ns (C1), 100 ns (C2), and 200 ns (C3). The corresponding results are summarized in Table 7 and illustrated in Figure 9. While Table 7 reports the dominant descriptors used for compact comparison, Figure 9 also includes the SatFull indicator, which remained negligible throughout the explored pulse-width range.

These results show that the baseline operating region does not collapse when the pulse width is increased within the explored range. As seen in the upper panel of Figure 9, the valid-event ratio remains effectively unchanged, staying at or very near unity across all three conditions. The lower panel shows that the saturation indicators remain low and that the number of occupied fine-code bins changes only slightly, from 39 to 37–38 bins. Taken together, these observations indicate that the selected export-compatible baseline is not excessively fragile with respect to moderate widening of the input pulse.

### 5.7. Safe Versus Aggressive Export Operating Points

**Figure 10:**
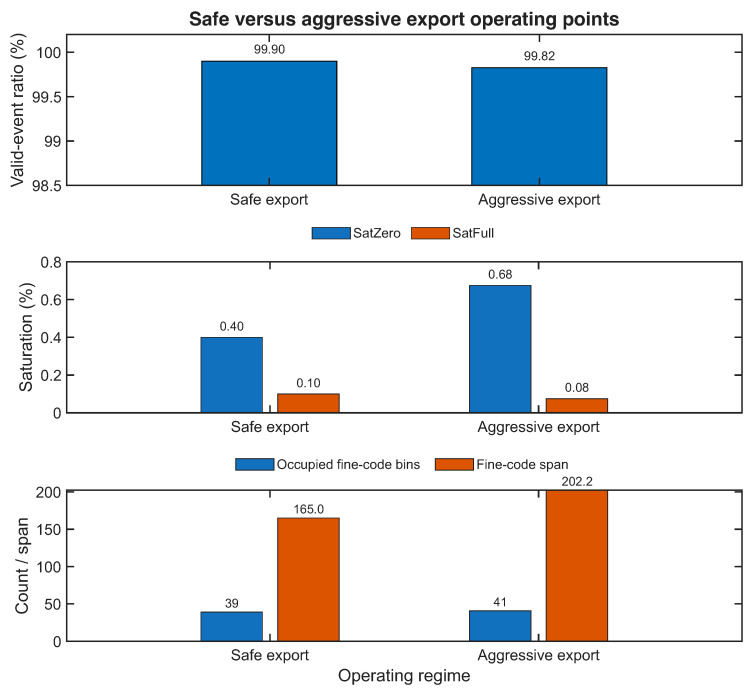
Comparison between the safe and aggressive export operating points. The upper panel reports the valid-event ratio, the middle panel compares the saturation indicators (SatZero and SatFull), and the lower panel summarizes the explored fine-code region through the number of occupied fine-code bins and the fine-code span. The aggressive operating point preserves a very high valid-event ratio while increasing the explored fine-code region, at the cost of only a modest increase in saturation-related indicators.

The next experimental objective was to determine if a more aggressive export operating point could preserve the high event validity of the safe baseline while extending the explored internal timing region. To address this, the representative safe export condition D1 (5 kHz, 20 ns) was compared against the aggressive export family formed by D2, G1, G2, and G3 (10 kHz, 10 ns). The comparison is summarized in Table 8 and visualized in Figure 10, where the valid-event ratio, the saturation indicators, and the fine-code exploration metrics are shown separately in order to preserve the readability of their different numerical scales.

Figure 10 confirms that the aggressive operating region does not collapse. The valid-event ratio remains very high in both regimes, the increase in saturation indicators is modest, and the occupied fine-code bins and span are broader at the aggressive point. The main trade-off is therefore not a collapse in validity but a modest increase in saturation indicators in exchange for a more widely explored fine-code region. The transition between these two anchor points is examined next through an intermediate export-frequency sweep.

### 5.8. Intermediate Export-Frequency Sweep and Operating Envelope

The comparison between the safe and aggressive export regimes establishes that the front-end can be operated under both conservative and more exploratory UART-compatible conditions. However, these two anchor points alone do not fully describe how the export-visible behavior evolves between them. To clarify that transition, an additional intermediate sweep was carried out at a fixed short pulse width while progressively increasing the export frequency between the safe and aggressive regimes.

In this additional experiment, the pulse width was kept fixed in the short-pulse regime while the event frequency was stepped through intermediate conditions between 5 kHz and 10 kHz. The purpose of this sweep was not to identify a single abrupt export limit, but rather to determine if the degradation of export-visible behavior, if any, emerges gradually or abruptly as the operating point moves from the safe regime toward the aggressive one. The same metrics used throughout the UART campaign were retained here as well, namely the valid-event ratio, the dominant saturation indicator, and two complementary descriptors of fine-code exploration: the number of occupied fine-code bins and the fine-code span. Here again, the fine-code span is used only as an empirical descriptor of how widely the processed valid-event population extends within the corresponding analysis dataset, rather than as a calibrated statement about the direct linear range of the exported 7-bit fine field.

**Figure 11:**
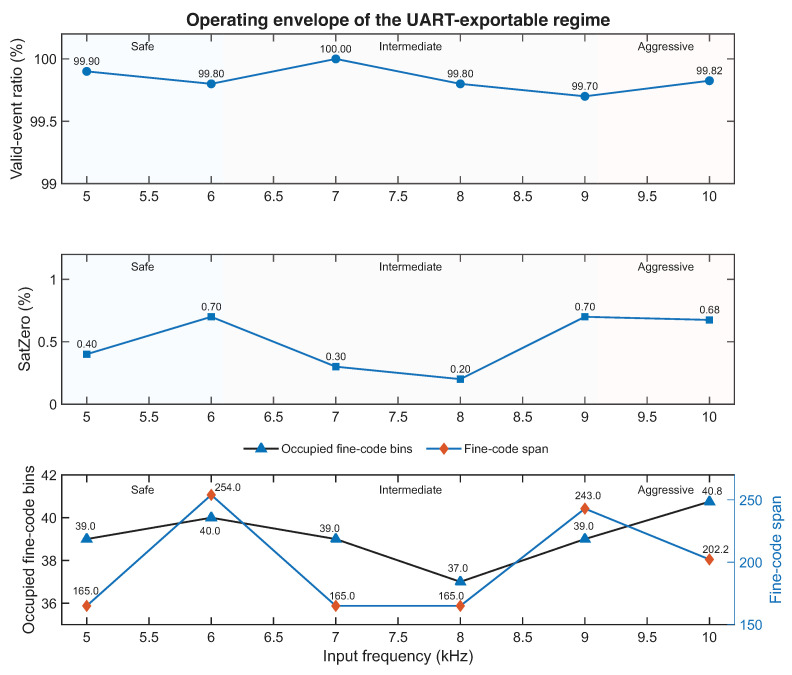
Operating-envelope summary of the UART-exportable regime. The three panels share the safe, intermediate, and aggressive operating regions highlighted in the background. The upper panel reports the valid-event ratio, the middle panel shows the evolution of zero-end saturation, and the lower panel summarizes fine-code exploration through the occupied fine-code bins (left axis) and the fine-code span (right axis). Together, these results show that the transition from the conservative export point to the more exploratory regime remains experimentally interpretable across the explored frequency range.

Figure 11 summarizes the resulting operating envelope. The background shading highlights the safe, intermediate, and aggressive regions across all three panels. The upper panel reports the valid-event ratio, the middle panel shows the evolution of zero-end saturation, and the lower panel summarizes fine-code exploration through the occupied-bin count and the fine-code span, which are shown separately to preserve the readability of both quantities despite their different numerical scales.

The operating-envelope view shows that the export-compatible behavior does not admit a binary “working/not working” interpretation. The valid-event ratio remains high, and zero-end saturation stays modest across the full range. Fine-code exploration does not evolve monotonically—both the occupied-bin count and the span fluctuate while remaining interpretable—consistent with the combined influence of the timing core, the stimulus, and the export chain. The sweep, therefore, connects the baseline, the intermediate points, and the aggressive endpoint into a coherent picture: degradation is gradual rather than abrupt, and the UART-visible limit is shaped by the full measurement chain rather than by a collapse of the internal timing core.

### 5.9. Integrated Interpretation of Internal and Export Modes

The individual result blocks gain additional meaning when considered together. The ILA mode shows that the internal timing front-end remains coherent under MHz-range short-pulse excitation, whereas the UART campaign identifies a practical host-visible operating space organized into safe, intermediate, and aggressive regions. With the addition of the intermediate export-frequency sweep, the transition between these regions can be interpreted experimentally rather than only conceptually.

In particular, Figure 11 shows that the practical UART-visible regime is not separated from the more exploratory region by an abrupt behavioral discontinuity. Instead, the measured valid-event ratio, saturation behavior, and fine-code exploration remain interpretable as the operating point moves from the conservative export region toward the aggressive endpoint. This observation supports the view that the practical export limit is shaped by the complete measurement chain rather than by a sudden collapse of the internal timing core itself.

Taken together, the reported measurements support a consistent interpretation of the proposed architecture as an experimentally characterized event-timing subsystem with distinct internal and export-visible operating regimes. This interpretation is developed further in the following discussion.

## 6. Discussion

### 6.1. System-Level Interpretation of the Front-End

The experimental results confirm that the proposed design is more usefully interpreted as a sensing-oriented timing front-end than as a carry-chain interpolation macro. Conventional TDC figures such as minimum time bin, RMS precision, and calibrated linearity remain important, but they are not sufficient for a front-end that must operate under asynchronous excitation, classify abnormal events, interface with a constrained export path, and support reproducible host-side analysis. The present work is therefore application-motivated and sensing-oriented but not a full detector-integrated sensor demonstration: it is a detector-independent timing front-end whose design choices are motivated by constraints common in SPAD-based time-resolved sensing workflows.

### 6.2. Internal Operating Capability Versus Export-Compatible Operation

A central conceptual result of the paper is the distinction between the internal operating capability of the timing front-end and the more restrictive regime that remains compatible with host-visible export. The measurements show that these two regimes are related but not identical: the ILA-based observations confirm coherent internal behavior under MHz-range short-pulse excitation, whereas the UART-based campaign defines the operating space in which large event sets can be exported and analyzed reproducibly. In this sense, the host-visible regime is constrained by the complete measurement chain, whereas the internal timing core remains functional in a substantially faster regime.

### 6.3. Meaning of the Safe and Aggressive Operating Regions

The results identify 5 kHz/20 ns as a safe export operating point. The usefulness of this point lies not in being the most aggressive condition the system can tolerate, but in combining several desirable properties simultaneously: very high valid-event ratio, negligible abnormal-event rates, reproducible packet capture, and consistent occupation of a meaningful fine-code subset. In other words, it is a condition that can be trusted for repeated automated acquisition.

This is an important practical result because sensing experiments often require long captures, repeated acquisitions, or parameter sweeps in which the user cannot afford to revalidate the measurement chain manually at every step. A front-end that behaves well only in isolated hand-tuned conditions is of limited value. The baseline results presented here instead indicate that the proposed front-end can sustain a quantitatively useful and reproducible operating regime over repeated runs and over time-separated acquisitions.

The identification of a more aggressive operating region at 10 kHz/10 ns adds a second layer to the experimental picture. This result shows that the front-end is not limited to a single conservative regime. Rather, it can also operate under a more demanding export condition that preserves high event validity while extending the explored fine-code range. The aggressive regime is not claimed to be universally better: the measurements show a modest increase in saturation-related indicators, which is entirely consistent with the intuition that a shorter-pulse, higher-rate condition should impose tighter constraints on the front-end. However, the degradation remains small compared with the gain in explored fine-code range, which makes the aggressive point experimentally meaningful rather than marginal.

Taken together, the safe and aggressive conditions show that the front-end is better understood in terms of a useful operating space than in terms of a single “best” point. This interpretation is reinforced by the intermediate sweep, which shows that the transition between conservative and more exploratory export regimes is gradual rather than abrupt.

### 6.4. Interpretation of the Pulse-Width Sweep

The pulse-width sweep at a fixed 5 kHz deserves a careful reading. Before the final campaign, it would have been reasonable to expect a narrative in which increased pulse width rapidly destroys the quality of the TDL observation. The measurements do not support such a strong statement in the 20 ns to 200 ns interval explored here. Instead, the front-end remained largely robust, with negligible change in the valid-event ratio and only small variations in saturation statistics and occupied fine-code bins.

This does not mean that pulse width is unimportant. On the contrary, pulse width clearly played a central role in selecting the useful operating region during exploratory work, and the short-pulse regime remained the preferred one for internal ILA validation. What the results show is more nuanced: once the export-compatible baseline has been identified at 5 kHz, the front-end does not collapse when the pulse is widened moderately within the explored range.

From the standpoint of sensing-oriented instrumentation, this is a useful rather than disappointing result. Robustness under moderate stimulus variation is often more valuable than a fragile optimum. The pulse-width sweep therefore contributes positively to the paper even though it does not support an extreme failure-boundary narrative. It shows that the baseline operating point is not excessively brittle, which strengthens the argument that it can serve as a practical measurement condition.

A related implication is that the robustness observed here should not be confused with formal linearity preservation. The pulse-width sweep shows that the selected export-compatible regime remains usable under moderate stimulus variation, but it does not by itself imply that the fine-time mapping is invariant or calibration-free. In an FPGA TDL, moderate changes in the excitation regime may leave event validity largely unchanged while still altering the detailed occupation of fine bins. This distinction is important for interpreting the present paper correctly: the sweep supports the robustness of front-end usability, not a full metrological invariance claim.

### 6.5. Relevance to Time-Resolved Sensing Workflows

The broader relevance of these results lies in their fit with the practical needs of time-resolved sensing. Recent discussions in SPAD and FLIM instrumentation emphasize that system value is often determined by the joint behavior of detector physics, timing electronics, throughput, and data conditioning rather than by a single isolated timing figure [7,16,21]. In SPAD-based ToF, TCSPC, FLIM, and other event-oriented acquisition chains, a timing front-end must do more than interpolate time finely. It must also sustain valid operation under asynchronous event arrival, preserve interpretability of the timestamp stream, and provide enough observability that invalid cases can be identified and managed. These requirements become particularly visible in systems where dead time, throughput, and bias introduced by invalid events can distort the measurement result if left untreated [3,5,6,27,28,29].

The proposed front-end does not claim to replace application-specific ASIC imagers or highly integrated photon-counting systems. Rather, it occupies a different but still relevant niche: it provides a flexible and accessible timing subsystem that can be embedded into a sensing-oriented laboratory workflow. In such workflows, it is often more useful to have a front-end whose internal behavior can be validated, whose export path can be trusted, and whose operating points are experimentally justified than to have a nominally finer but less observable black-box timing block.

This is why the paper emphasizes methodology as much as architecture. The dual-mode strategy is not a peripheral convenience; it is part of what makes the front-end useful for sensing-oriented experimentation. It allows the same hardware to be understood both as an internally fast event detector and as a conservative, host-compatible measurement subsystem.

This connection can be made more concrete in SPAD-oriented terms. In practical TCSPC and related photon-timing workflows, the timing contribution of the front-end should remain smaller than, or at least not overwhelmingly dominate, the detector-side temporal uncertainty if the overall system is to preserve useful timing resolution. In many CMOS SPAD technologies, detector-side timing jitter spans the range from a few tens of picoseconds to several hundreds of picoseconds, depending on device structure, process, and operating conditions. Likewise, dead-time-aware handling is directly relevant because SPAD-based chains are themselves shaped by detector recovery and event-spacing constraints, often in the tens-of-nanoseconds range depending on the device and readout organization. From that perspective, the present front-end is not offered as a detector-integrated SPAD readout but as a detector-independent timing subsystem whose event qualification, hold-off handling, and export-aware characterization are aligned with constraints that are common in SPAD-based laboratory timing workflows. In the present experiments, the configured 320 ns hold-off is conservative with respect to the 5 kHz to 10 kHz UART campaign and therefore is not a practically limiting factor at the reported operating points. Its significance lies instead in showing that the front-end already includes a mechanism compatible with scenarios in which events can occur much closer in time, as is common in detector-driven timing chains. Accordingly, in the present paper, the dead-time-aware block should be understood primarily as an architectural provision aligned with detector-driven and higher-rate asynchronous timing scenarios, rather than as a feature that is quantitatively stressed by the low-rate UART operating points used in the reported export campaign.

### 6.6. Relationship to the FPGA-TDC Literature

Relative to the FPGA-TDC literature, the present contribution is not positioned by raw-resolution claims. Prior work has already demonstrated that carry-chain TDLs can achieve impressive timing performance under careful design and calibration [30,31,33,34,36,38]. What the present paper adds is an experimentally grounded way of organizing such a front-end for sensing-oriented use: the novelty lies in the integration of event qualification, dual-mode characterization, and export-aware operating-point selection. This comparative interpretation is quantified in the tables below.

### 6.7. Comparative Positioning with Prior FPGA-TDC and Sensing-Oriented Front-Ends

Table 9 positions the present work against representative FPGA-based TDC implementations. Rather than claiming the best absolute resolution, the comparison highlights the dimensions most distinctive to this contribution: explicit event-quality qualification, dead-time-aware handling, export-compatible operation, ILA-based internal observability, and structured characterization of safe, intermediate, and aggressive operating regions.

As Table 9 makes clear, the contribution combines a low-cost Artix-7 implementation with a dual-mode validation strategy—internal MHz-range ILA observation and repeatable host-visible UART export, in which trustworthiness, observability, and export compatibility are treated as first-class design objectives alongside timing granularity.

To complement the qualitative positioning of Table 9, Table 10 summarizes a compact set of quantitative indicators that are directly relevant to the present implementation, namely nominal within-clock-cycle granularity, estimated functional implementation cost, and the scope of reported linearity-related characterization. The purpose of this table is not to infer strict one-to-one comparability across architectures with different channel counts, calibration procedures, FPGA families, and validation methodologies, but to place the present Artix-7 front-end within a more explicit quantitative context.

As shown in Table 9, several prior FPGA-TDC works report stronger raw timing metrics, including finer effective resolution, lower RMS precision, higher channel count, or more advanced calibration procedures [30,31,33,34,38]. By contrast, the present front-end contributes a different kind of result: a compact and experimentally observable timing subsystem in which internal correctness, event qualification, export compatibility, and operating-envelope characterization are treated as first-class design objectives. This makes the work particularly relevant for time-resolved sensing chains and laboratory instrumentation scenarios in which trustworthy and reproducible event streaming matters alongside timing granularity.

### 6.8. Limitations and Scope

The interpretation above should be balanced with a clear statement of limitations. First, the present paper does not provide a full calibration-heavy metrological characterization in the sense of detailed DNL/INL extraction, single-shot RMS precision evaluation under a dedicated swept-delay setup, or formal temperature/voltage drift compensation. These are important topics and are strongly represented in prior FPGA-TDC literature, but they were not the primary focus of the present campaign.

Second, the ILA mode, while extremely useful for internal validation, is not intended as a substitute for long statistical acquisition. Its capture depth, trigger dependence, and structured observability make it ideal for internal behavior verification but less appropriate as the sole basis for large-scale quantitative claims. That is precisely why the paper treats ILA and UART as complementary rather than interchangeable.

Third, the export mode is constrained by the UART path and by the external signal generator’s duty-cycle granularity. These are real limitations of the experimental setup. However, they do not invalidate the results; instead, they define the regime in which the present study is honest and reproducible. One of the strengths of the work is that these constraints are made explicit rather than hidden.

Finally, the conclusions of the paper should be interpreted as applying to the demonstrated front-end architecture and workflow rather than as a universal statement about all FPGA-based timing systems. The goal is to provide a robust, experimentally justified case study and methodology, not to claim a fully general theory of export-limited TDC operation.

### 6.9. Implications for Future Work

The most immediate extension of the present work would be to combine the current dual-mode methodology with a more formal timing-metrology campaign. In practical terms, this would mean preserving the internal-versus-export distinction established here while adding a calibrated swept-delay setup capable of supporting bin-width estimation, DNL/INL extraction, and direct precision measurements. Such an extension would make it possible to connect the present operating-point-based analysis with the classical performance descriptors of the TDC literature.

A second direction would be to strengthen the export path. The present results already show that the internal core can operate in a faster regime than the UART-friendly one. Replacing or augmenting the export interface with a higher-bandwidth path would therefore allow future work to probe the boundary between internal capability and sustained host-visible throughput more directly.

A third promising direction lies in application-level integration. Since the front-end already exposes valid-event information and supports conservative versus aggressive operating-point selection, it could be coupled naturally to event-driven sensing demonstrators in which timestamp trustworthiness matters as much as nominal timing granularity. Examples include SPAD-based laboratory demonstrators, pulsed radiation sensor front-ends, or asynchronous event-classification chains in which timing and event validity must be considered jointly.

## 7. Conclusions

This work has presented and experimentally characterized an FPGA-based edge-event timing front-end for time-resolved sensing and event-driven measurement scenarios. The resulting system combines carry-chain-based fine interpolation, coarse–fine timestamp formation, explicit event-quality assessment, dead-time-aware handling, and lightweight UART-compatible export within a unified sensing-oriented timing workflow.

The results support a dual interpretation of the front-end. On the one hand, the ILA-based validation mode shows that the internal timing core exhibits coherent internal behavior under MHz-range short-pulse excitation, with valid internal events, stable fine-code behavior, and clean status indicators. On the other hand, the UART-based campaign identifies a practically useful export-visible operating space organized into safe, intermediate, and aggressive regimes. Within that space, the front-end maintains high event validity, low saturation, and nontrivial fine-code exploration, while the operating-envelope analysis shows that the transition from conservative to more exploratory conditions remains interpretable across the explored range and is not associated with catastrophic degradation of export-visible behavior.

The final comparative positioning clarifies the scope of the contribution. The value of the proposed front-end lies in showing that a low-cost FPGA-based timing subsystem can be made experimentally meaningful through the combination of internal observability, explicit event qualification, export-aware design, and structured operating-region characterization. Overall, the manuscript shows that the proposed architecture can be interpreted as a compact event-timing subsystem whose behavior is characterized across complementary internal and export-visible regimes, providing a useful basis for future work toward richer export interfaces, denser operating-envelope exploration, more formal timing-metrology extensions, and more application-specific time-resolved sensing chains. 

## Figures and Tables

**Table 1:** Representative prior work used to position the proposed front-end with respect to FPGA-based TDC implementations and time-resolved sensing systems.

Work	Platform	Channels	Timing Emphasis	Distinctive Feature	Main Relevance to This Work
Favi and Charbon, 2009 [30]	65 nm FPGA	1	Fine FPGA timing	Early carry-chain benchmark	Historical FPGA-TDC reference for carry-chain timing.
Bayer and Traxler, 2011 [31]	FPGA	48	High precision	Multichannel architecture	Representative multichannel precision FPGA-TDC implementation.
Liu and Wang, 2015 [33]	Kintex-7	128	High throughput and precision	Large channel count	High-performance FPGA-TDC reference beyond the low-cost single-channel scope of this work.
Chen et al., 2017 [34]	28 nm FPGA	N/A	Low nonlinearity	Embedded bin-width calibration	Representative calibration-oriented FPGA-TDC work.
Song et al., 2020 [38]	60 nm FPGA	N/A	Fine precision	Real-time temperature correction	Reference for robustness-oriented timing improvement.
Garzetti et al., 2024 [39]	FPGA system	N/A	Timestamp handling	Dead time and configurable timestamp management	Closest prior work in terms of system-level timing management.
Chen et al., 2025 [40]	Artix-7	1	Heterogeneous TDL	Same-family device relevance	Closest same-family FPGA context for Artix-7 carry-chain timing.
Niclass et al., 2013 [24]	CMOS dToF sensor	Array	Long-range depth timing	Correlation-based CMOS ToF depth sensor	Application-side timing relevance for SPAD-based ranging systems.
Dutton et al., 2015 [25]	CMOS SPAD/TCSPC sensor	Array	Histogramming TDC	Very high in-sensor timing throughput	Relevant SPAD/TCSPC timing-chain reference.
Piron et al., 2021 [5]	SPAD ToF review	—	ToF sensor architectures	Broad direct/indirect ToF comparison	System-level sensing context for time-resolved applications.
Bruschini et al., 2019 [3]	SPAD imaging review	—	Time-resolved optical sensing	Review and outlook	Broad SPAD-based sensing context motivating detector-side timing chains.
This work	Basys 3/Artix-7	1	Sensing-oriented event timing	Event qualification, ILA observability, dual-mode characterization	Low-cost FPGA timing front-end oriented to experimentally observable and export-aware operation in time-resolved sensing workflows.

**Table 2:** Functional summary of the key event-processing blocks in the proposed front-end. The table complements Figure 1 and Figure 2 by explicitly listing the main inputs, outputs, and functional role of the blocks that govern event interpretation, qualification, and acceptance.

Block	Main Inputs	Main Outputs	Main Function
Coherent state capture	TDL propagation state, observation instant	Latched raw tap vector	Samples the asynchronous TDL state into a stable digital representation that can be interpreted by subsequent logic.
Cleanup logic	Latched raw tap vector	Cleaned thermometer-like representation, anomaly indicators	Filters or rejects non-ideal transition patterns and produces a more interpretable edge representation for internal analysis and fine-time extraction.
Fine encoder	Cleaned thermometer-like representation	event_fine[6:0]	Determines the fine-time position associated with the accepted transition pattern and exports it as the encoded fine event field.
Event-quality classification	Raw/corrected transition structure, encoded fine result	fine_valid, sat_zero, sat_full, multi_edge	Assigns event-validity and anomaly flags so that the exported timestamp stream can distinguish acceptable events from suspicious or pathological ones.
Dead-time-aware handling	Qualified event candidate, hold-off state	Accepted event strobe, blocked event condition	Implements the event-acceptance policy by suppressing events that arrive too closely after a previously accepted event.
UART packetizer	Accepted coarse–fine event, flags	UART frame fields	Packages accepted events into a host-visible export format including timing information, quality indicators, and integrity fields.

**Table 3:** Implementation cost on the Basys 3/Artix-7 platform. The debug-enabled build includes the ILA core and debug hub used during internal validation. The functional-design values are estimated by excluding those debug blocks from the reported post-implementation hierarchy and are included to reflect the practical cost of the sensing front-end itself.

Resource	Available	Debug-Enabled	Functional (est.)	Utilization (est.)
Slice LUTs	20,800	9987	∼1328	∼6.4%
Slice Registers	41,600	13,008	∼543	∼1.3%
Occupied Slices	8150	3792	∼381	∼4.7%
Block RAM Tiles	50	47	0	0%
BUFGCTRL	32	2	1	∼3.1%

Note: The functional-design values are estimated from the post-implementation hierarchical utilization by excluding the ILa and debug blocks.

**Table 4:** Summary of the experimental matrix used in the measurement campaign.

Block	Mode	Representative Conditions	Purpose	Main Metrics
A	ILA internal validation	∼1.00 MHz/1.00%, ∼ 1.01 MHz/1.01% (short pulses)	Verify coherent internal event detection, thermometer behavior, fine-code stability, and clean flags under MHz-range excitation	Internal valid events, saturation/multi-edge occurrence, occupied fine-code range
B	UART export baseline	5 kHz, 20 ns	Establish the safe export-compatible operating point	Accepted packets, valid-event ratio, saturation, multi-edge rate, fine-code range, occupied bins
C	UART width sweep	5 kHz with 20 ns, 100 ns, 200 ns	Evaluate robustness of the baseline against pulse-width variation	Valid-event ratio, saturation, occupied bins, fine-code range
D	UART safe vs aggressive	5 kHz/ 20 ns versus 10 kHz/ 10 ns	Compare conservative and more exploratory export-compatible operating points	Valid-event ratio, saturation, occupied bins, fine-code span
E	UART intermediate sweep	Intermediate frequencies between 5 kHz and 10 kHz at short pulse width	Determine if export-visible degradation evolves gradually or abruptly	Valid-event ratio, saturation, occupied bins, fine-code span
F	UART repeatability/temporal stability	Repeated baseline runs across the same and later sessions	Verify that the baseline is reproducible and stable in time	Accepted packets, valid-event ratio, saturation, occupied bins, fine-code range

**Table 5:** Aggregate summary of the safe export baseline family (5 kHz, 20 ns), including the initial, extended-repeatability, and temporal-stability runs. Statistics are computed across 12 runs (B1–B3, R1–R5, T1–T3). Min and max values characterize the full observed range rather than only the central tendency.

Metric	Mean	Std. Dev.	Min	Max
Number of runs	12	—	—	—
Valid-event ratio (%)	99.92	0.21	99.30	100.00
Saturation at zero (%)	0.33	0.28	0.00	0.90
Saturation at full (%)	0.06	0.07	0.00	0.20
Multi-edge ratio	negligible across all runs			
Occupied fine-code bins	38.17	2.86	35	44
Received packets per run	1000	0	1000	1000

**Table 6:** Preliminary metrological indicators extracted from dedicated filtered UART captures.

Metric	Measured Value
Occupied fine-code bins	64
Samples	120,000
Max DNL (LSB)	0.210
Min DNL (LSB)	−0.206
INL peak-to-peak (LSB)	1.164
RMS fine-code dispersion (bins)	0.482
RMS fine-time dispersion, nominal (ps)	37.636
RMS fine-time dispersion, code-density-derived (ps)	88.087

**Table 7:** Pulse-width robustness results at fixed 5 kHz event rate.

Condition	Pulse Width	Valid (%)	SatZero (%)	NumBins
C1	20 ns	100.0	0.3	37
C2	100 ns	100.0	0.3	38
C3	200 ns	100.0	0.4	37

**Table 8:** Comparison between the safe export operating point and the aggressive export family.

Metric	Safe Export	Aggressive Export
Representative condition	5 kHz, 20 ns	10 kHz, 10 ns
Number of runs	1	4
Mean valid-event ratio	99.9%	99.83%
Mean saturation at zero	0.3%	0.67%
Mean saturation at full	0.0%	0.08%
Mean multi-edge ratio	negligible	very low
Mean occupied fine-code bins	38.0	40.75
Fine-code range tendency	narrower	broader

**Table 9:** Final comparative positioning of the proposed front-end with respect to representative FPGA-TDC works.

Work	Platform	Ch.	Timing Focus	Calibration/ Linearity	Event Qualification	Export/ Visibility	Main Distinguishing Contribution
Favi and Charbon [30]	65 nm FPGA	1	Fine resolution	Not central	No	No	Early high-resolution FPGA-TDC benchmark.
Bayer and Traxler [31]	FPGA	48	<10 ps RMS class	Not central	No	No	High-channel-count precision timing.
Liu and Wang [33]	Kintex-7	128	High throughput/low RMS	Not central	No	No	Scaled multichannel high-performance FPGA TDC.
Chen et al. [34]	28 nm FPGA	N/A	Missing-code-free behavior	Strong	No	No	Embedded calibration with low nonlinearity.
Song et al. [38]	60 nm FPGA	N/A	8.8 ps RMS class	Temperature correction	No	Limited	Robustness against thermal drift.
Garzetti et al. [39]	FPGA system	N/A	Timestamp management	System-level	Partial	Moderate	Dead time and configurable timestamp management.
This work	Basys 3/Artix-7	1	Coarse–fine event timing under export constraints	Operating-envelope characterization	Yes	Yes	Dual-mode validation, event qualification, dead-time-aware handling, and export-regime characterization.

**Table 10:** Compact quantitative positioning of the present work with respect to representative FPGA-TDC references. Reported values are taken directly from the cited works when available. “Nominal granularity” for this work refers to the average within-clock-cycle scale implied by the 100 MHz clock and 128-tap TDL, and should not be interpreted as a calibrated bin width.

Work	Timing Figure	Implementation/Scale	Linearity Characterization
Favi and Charbon [30]	17 ps TDC class	1 channel, 65 nm FPGA	Not central
Bayer and Traxler [31]	<10 ps RMS class	48 channels, FPGA	Not central
Liu and Wang [33]	<10 ps RMS class	128 channels, Kintex-7	Not central
Chen et al. [34]	Low nonlinearity/missing-code free	28 nm FPGA	Embedded calibration, DNL/INL oriented
Song et al. [38]	8.8 ps RMS class	60 nm FPGA	Temperature-aware correction
This work	∼78 ps nominal within-clock-cycle granularity; preliminary RMS and DNL/INL descriptors	∼1328 LUTs, ∼543 registers, ∼381 slices (functional estimate), 1 channel, Artix-7	Preliminary DNL/INL/RMS from dedicated metrological acquisition

## Data Availability

The original contributions presented in this study are included in the article. Further inquiries can be directed to the corresponding author.

## References

[1] Henzler S. (2010). Time-to-Digital Converters.

[2] Szyduczyński J., Kościelnik D., Miśkowicz M. (2023). Time-to-digital conversion techniques: A survey of recent experimental developments. Measurement.

[3] Bruschini C., Homulle H., Antolovic M.I., Burri S., Charbon E. (2019). Single-photon avalanche diode imagers in biophotonics: Review and outlook. Light Sci. Appl..

[4] Bronzi D., Villa F., Tisa S., Tosi A., Zappa F. (2016). SPAD figures of merit for photon-counting, photon-timing, and imaging applications: A review. IEEE Sens. J..

[5] Piron F., Morrison D., Yuce M.R., Redouté J.M. (2021). A review of single-photon avalanche diode time-of-flight imaging sensor arrays. IEEE Sens. J..

[6] Datta R., Heaster T.M., Sharick J.T., Gillette A.A., Skala M.C. (2020). Fluorescence lifetime imaging microscopy: Fundamentals and advances in instrumentation, analysis, and applications. J. Biomed. Opt..

[7] Villa F., Severini F., Madonini F., Zappa F. (2021). SPADs and SiPMs arrays for long-range high-speed light detection and ranging (LiDAR). Sensors.

[8] Padmanabhan P., Zhang C., Charbon E. (2019). Modeling and analysis of a direct time-of-flight sensor architecture for LiDAR applications. Sensors.

[9] Sesta V., Severini F., Villa F., Lussana R., Zappa F., Nakamuro K., Matsui Y. (2021). Spot tracking and TDC sharing in SPAD arrays for TOF LiDAR. Sensors.

[10] Morsy A., Kuijk M. (2024). Correlation-assisted pixel array for direct time of flight. Sensors.

[11] Miyazawa R., Shirakawa Y., Mars K., Yasutomi K., Kagawa K., Aoyama S., Kawahito S. (2023). A time-of-flight image sensor using 8-tap P-N junction demodulator pixels. Sensors.

[12] Wang Z., Yin X., Tu N., Li J., Li L. (2024). A 64 × 128 3D-stacked SPAD image sensor for low-light imaging. Sensors.

[13] Fu C., Liang J., Li Y., Yang J. (2020). Three-dimensional imaging via time-correlated single-photon counting technology. Appl. Sci..

[14] Chunnilall C.J., Degiovanni I.P., Kück S., Müller I., Sinclair A.G. (2014). Metrology of single-photon sources and detectors: A review. Opt. Eng..

[15] Wei L., Yan W., Ho D. (2017). Recent advances in fluorescence lifetime analytical microsystems: Contact optics and CMOS time-resolved electronics. Sensors.

[16] Park J., Gao J., Marcu L. (2024). Advancements in fluorescence lifetime imaging microscopy instrumentation: Towards high-speed and 3D. Curr. Opin. Solid State Mater. Sci..

[17] Cova S., Lacaita A., Ghioni M., Ripamonti G., Louis T.A. (1989). 20-ps timing resolution with single-photon avalanche diodes. Rev. Sci. Instrum..

[18] Cova S., Ghioni M., Lacaita A., Samori C., Zappa F. (1996). Avalanche photodiodes and quenching circuits for single-photon detection. Appl. Opt..

[19] Zappa F., Tisa S., Tosi A., Cova S. (2007). Principles and features of single-photon avalanche diode arrays. Sens. Actuators A.

[20] Ghioni M., Gulinatti A., Rech I., Zappa F., Cova S. (2007). Progress in silicon single-photon avalanche diodes. IEEE J. Sel. Top. Quantum Electron..

[21] Acconcia G., Ceccarelli F., Gulinatti A., Rech I., Zappa F. (2023). Timing measurements with silicon single photon avalanche diodes: Principles and perspectives [Invited]. Opt. Express.

[22] Real D., Calvo D. (2024). Low-resource time-to-digital converters for field programmable gate arrays: A review. Sensors.

[23] Machado R., Cabral J., Alves F.S. (2019). Recent developments and challenges in FPGA-based time-to-digital converters. IEEE Trans. Instrum. Meas..

[24] Niclass C., Soga M., Matsubara H., Kato S. (2013). A 100-m range 10-frame/s 340 × 96-pixel time-of-flight depth sensor in 0.18-μm CMOS. IEEE J. Solid-State Circuits.

[25] Dutton N.A.W., Gnecchi S., Parmesan L., Holmes A.J., Rae B.R., Grant L., Henderson R.K. (2015). A time-correlated single-photon-counting sensor with 14GS/s histogramming time-to-digital converter. 2015 IEEE International Solid-State Circuits Conference (ISSCC) Digest of Technical Papers, San Francisco, CA, USA, 22–26 February 2015.

[26] Krstajć N., Poland S., Levitt J., Walker R., Erdogan A., Simon A.B., Henderson R.K. (2015). 0.5 billion events per second time correlated single photon counting using CMOS SPAD arrays. Opt. Lett..

[27] Isbaner S., Karedla K., Ruhlandt K., Barczyk M.H., Pieper J., Gregor A.C., Enderlein A., Lamb D.C. (2016). Dead-time correction of fluorescence lifetime measurements and fluorescence lifetime imaging. Opt. Express.

[28] Rapp J., Ma Y., Dawson R.M.A., Goyal V.K. (2019). Dead time compensation for high-flux ranging. IEEE Trans. Signal Process..

[29] Rapp J., Ma Y., Dawson R.M.A., Goyal V.K. (2021). High-flux single-photon lidar. Optica.

[30] Favi C., Charbon E. (2009). A 17 ps time-to-digital converter implemented in 65 nm FPGA technology. Proceedings of the ACM/SIGDA International Symposium on Field-Programmable Gate Arrays, Monterey, CA, USA, 22–24 February 2009.

[31] Bayer E., Traxler M. (2011). A high-resolution (<10 ps RMS) 48-channel time-to-digital converter (TDC) implemented in a field
programmable gate array (FPGA).. IEEE Trans. Nucl. Sci..

[32] Wang H., Zhang M., Yao Q. (2013). A new realization of time-to-digital converters based on FPGA internal routing resources. IEEE Trans. Ultrason. Ferroelectr. Freq. Control.

[33] Liu C., Wang Y. (2015). A 128-channel, 710 M samples/second, and less than 10 ps RMS resolution time-to-digital converter implemented in a Kintex-7 FPGA. IEEE Trans. Nucl. Sci..

[34] Chen H., Zhang Y., Li D.D. (2017). A low nonlinearity, missing-code free time-to-digital converter based on 28-nm FPGAs with embedded bin-width calibrations. IEEE Trans. Instrum. Meas..

[35] Tontini A., Gasparini L., Pancheri L., Passerone R. (2018). Design and characterization of a low-cost FPGA-based TDC. IEEE Trans. Nucl. Sci..

[36] Chen H., Li D.D. (2019). Multichannel, low nonlinearity time-to-digital converters based on 20 and 28 nm FPGAs. IEEE Trans. Ind. Electron..

[37] Kwiatkowski P., Szplet R. (2020). Efficient implementation of multiple time coding lines-based TDC in an FPGA device. IEEE Trans. Instrum. Meas..

[38] Song Z., Zhao Z., Yu H., Yang J., Zhang X., Sui T., Xu J., Xie S., Huang Q., Peng Q. (2020). An 8.8 ps RMS resolution time-to-digital converter implemented in a 60 nm FPGA with real-time temperature correction. Sensors.

[39] Garzetti F., Bonanno G., Lusardi N., Ronconi E., Costa A., Geraci A. (2024). New high-rate timestamp management with real-time configurable virtual delay and dead time for FPGA-based time-to-digital converters. Electronics.

[40] Chen R., Chen P., Li K., Liu H. (2025). Heterogeneous tapped delay-line time-to-digital converter on Artix-7 FPGA. Sensors.

